# Dexamethasone for Inpatients With COVID-19 in a National Cohort

**DOI:** 10.1001/jamanetworkopen.2023.8516

**Published:** 2023-04-17

**Authors:** Ahmad Mourad, Dylan Thibault, Thomas L. Holland, Siyun Yang, Allison R. Young, Shanna A. Arnold Egloff, Laine E. Thomas

**Affiliations:** 1 Department of Medicine, Division of Infectious Diseases, Duke University Medical Center, Durham, North Carolina; 2 Duke Clinical Research Institute, Durham, North Carolina; 3 Meta Platforms, Inc, Seattle, Washington; 4 Biobot Analytics, Cambridge, Massachusetts; 5 HCA Healthcare Research Institute, Brentwood, Tennessee; 6 Department of Biostatistics and Bioinformatics, Duke University, Durham, North Carolina

## Abstract

**Importance:**

Limited effective therapeutics are available to hospitalized patients with COVID-19. Clinical trials and observational studies have shown varying effects of systemic corticosteroids, including dexamethasone, in hospitalized patients with COVID-19, with limited descriptions of important patient subgroups.

**Objective:**

To examine the clinical use of dexamethasone for hospitalized patients with COVID-19 respiratory illness and to explore the heterogeneity of treatment outcomes across different subgroups.

**Design, Setting, and Participants:**

This is a retrospective, propensity score–weighted cohort study of adult patients hospitalized for at least 48 hours for COVID-19 respiratory illness between July 1, 2020, and October 31, 2021, at a large health care network of 156 hospitals across the US. Data analysis was performed from March 2022 to February 2023.

**Exposures:**

Systemic dexamethasone administered within 48 hours of either admission or escalation in oxygen support.

**Main Outcomes and Measures:**

All-cause in-hospital mortality or discharge to hospice.

**Results:**

A total of 80 699 patients who met the eligibility criteria were identified (median [IQR] age, 64 [52-76] years; 37 606 women [46.6%]); 13 230 patients (16.4%) identified as Black, 49 222 (60.9%) as White, 18 247 (22.6%) as other race, and 20 340 (25.2%) as Hispanic ethnicity. Of these patients, 13 040 (16.2%) did not require supplemental oxygen within 48 hours of admission, 56 368 (69.8%) required supplemental oxygen, 7618 (9.4%) required noninvasive positive pressure ventilation (NIPPV), and 3673 (4.6%) required mechanical ventilation (MV) and/or extracorporeal membrane oxygenation (ECMO). After adjustment by propensity score overlap weighting, early use of dexamethasone was associated with reduction in a composite outcome of in-hospital mortality or discharge to hospice for patients receiving supplemental oxygen (aOR, 0.92; 95% CI, 0.86-0.98) and MV and/or ECMO (aOR, 0.82; 95% CI, 0.68-0.99). In contrast, all-cause inpatient mortality or discharge to hospice was not lower for patients who received dexamethasone in the no supplemental oxygen group (aOR, 0.90; 95% CI, 0.78-1.03) and in the NIPPV group (aOR, 0.87; 95% CI, 0.73-1.04). Importantly, patients with more comorbidities had greater benefit from dexamethasone use.

**Conclusions and Relevance:**

In this national multicenter cohort study of inpatients with COVID-19, early administration of dexamethasone was associated with significantly reduced odds of mortality or discharge to hospice in those requiring supplemental oxygen or MV and/or ECMO but not in those requiring no supplemental oxygen or NIPPV. These results support the continued use of systemic dexamethasone in patients hospitalized with COVID-19.

## Key Points

**Question:**

Among hospitalized patients with COVID-19 respiratory illness in the US, is the early administration of dexamethasone associated with improved in-hospital outcomes?

**Findings:**

In this national cohort study of 80 699 patients, after adjustment by propensity score overlap weighting, early dexamethasone use was associated with a statistically significant reduction in a composite outcome of in-hospital mortality or discharge to hospice for patients receiving supplemental oxygen or mechanical ventilation and/or extracorporeal membrane oxygenation.

**Meaning:**

These findings suggest that early dexamethasone use is associated with improved mortality or discharge to hospice among patients requiring supplemental oxygen or mechanical ventilation and/or extracorporeal membrane oxygenation.

## Teaser

This cohort study examines the use of dexamethasone in hospitalized patients with COVID-19 respiratory illness and explores the heterogeneity of treatment outcomes across different subgroups.

## Introduction

Despite millions of deaths globally from COVID-19 and thousands of trials exploring novel and repurposed drugs, few effective therapeutics are available to hospitalized patients with COVID-19.^1^ The National Institutes of Health COVID-19 treatment guidelines^1^ currently recommend using systemic corticosteroids as the cornerstone of therapy in hospitalized patients with COVID-19 who require supplemental oxygen. This recommendation is largely based on the practice-changing results of the Randomized Evaluation of COVID-19 Therapy (RECOVERY) trial.^2^ In the RECOVERY study, administration of 6 mg of dexamethasone daily for up to 10 days was associated with a reduction in 28-day mortality in patients receiving oxygen only (risk ratio, 0.82; 95% CI, 0.72-0.94) or mechanical ventilation (MV) (risk ratio, 0.64; 95% CI, 0.51-0.81).^2^

Although the RECOVERY^2^ platform was pragmatic, had a large sample size, and helped inform clinical management of patients with COVID-19 during a rapidly evolving pandemic, there were some limitations. Most notably, it was an open-label study with no placebo group and no defined standard of care at the time, several levels of supplemental oxygen support were grouped together, and there was high mortality rate in the control groups. Several other smaller clinical trials^3,4,5^ examining dexamethasone, as well as other systemic corticosteroids, did find a benefit but reported different effect sizes. Furthermore, we still lack a more detailed understanding of the outcomes of dexamethasone treatment in certain patient subgroups. Given that outcomes with COVID-19 vary by patient characteristics,^6^ understanding the differing effects of dexamethasone can increase therapeutic precision by defining which patient subgroups benefit most.

As such, we conducted a large, national, multicenter cohort study to examine and detail the clinical use of dexamethasone in hospitalized patients with COVID-19 respiratory illness and to explore the propensity-adjusted association with in-hospital outcomes. Furthermore, we aimed to examine the heterogeneity of treatment outcomes across different patient subgroups, including groups that were not described in clinical trials.

## Methods

The Duke University institutional review board reviewed and approved the study before it began. Informed consent was not obtained because the data were anonymous, in accordance with 45 CFR §46. This article conforms to the Strengthening the Reporting of Observational Studies in Epidemiology (STROBE) reporting guideline for cohort studies.

### Data

Study data were compiled by the COVID-19 Consortium of HCA Healthcare and Academia for Research Generation and included electronic health records for patients hospitalized with laboratory-confirmed SARS-CoV-2 infection between July 1, 2020, and October 31, 2021, at 156 HCA Healthcare–affiliated facilities across the US (eFigure 1 in Supplement 1).^7,8^ Derived data sets were compiled by HCA and included patient demographics, historical diagnosis, COVID-19 testing results, and COVID-19 care details throughout the hospital encounter (eFigure 2 in Supplement 1).

### Patient Selection

We selected adult patients (aged >17 years), hospitalized and alive for at least 48 hours, with an *International Statistical Classification of Diseases and Related Health Problems, Tenth Revision* diagnosis code for COVID-19, as well as respiratory illness during the same encounter. The level of oxygen support was categorized using a modified World Health Organization COVID-19 ordinal scale (eAppendix 1 in Supplement 1), and available data on liters per minute of supplemental oxygen delivered were used to stratify patients into 4 analytic cohorts on the basis of their daily supplemental oxygen requirement. We excluded patients with consecutive missing ordinal scale status and those who received dexamethasone before admission (eAppendix 2, eFigure 3, and eFigure 4 in Supplement 1). The eligible population was stratified into 4 cohorts: (1) no supplemental oxygen, (2) supplemental oxygen, (3) noninvasive positive pressure ventilation (NIPPV), and (4) MV and/or extracorporeal membrane oxygenation (ECMO). Patients were eligible for a given cohort if they were hospitalized at that level of oxygen support (immediately eligible) or stepped up to that level of oxygen support without previously starting systemic steroids. Patients were included in a given cohort at the first time of eligibility, regardless of changes to oxygen support that could arise during subsequent follow-up for outcomes. Patients were required to remain alive and under follow-up for 48 hours after initial eligibility, and patients who started a steroid other than dexamethasone within this window were excluded. The question of interest for each cohort was, for a hospitalized patient who immediately received or required escalation to a particular level of supplemental oxygen support, what was the association with outcome of starting dexamethasone within 48 hours vs not starting any steroid in that time?

### Variables and Outcomes

In each cohort, early dexamethasone or control status was determined within the initial 48-hour window of eligibility, and covariates were defined by the last value within that window before treatment. Follow-up for outcomes began at the end of the 48-hour window (time 0). Because this was an inpatient data set and 30-day mortality was not available for those discharged before 30 days of admission, discharge to hospice was added to all-cause in-hospital mortality to create our composite primary outcome of interest. This avoids categorization of hospital discharge to hospice as treatment success. Systemic dexamethasone was defined as oral or intravenous administration. Confounders were identified a priori by clinical input and review of the literature and included hospital characteristics, patient demographic characteristics, comorbidities, and measures of COVID-19 disease severity. Select variables of importance are displayed in Table 1, and the full set of variables is described in eAppendix 2, eTable 1, eTable 2, eTable 3, and eTable 4 in Supplement 1. Among patients receiving supplemental oxygen, low-flow oxygen was defined as 15 L/minute or less, and high-flow oxygen was defined as more than 15 L/minute, which would typically require advanced modes of delivery, such as high-flow, high-humidity nasal cannula, and would require a higher level of clinical care.

**Table 1.** Selected Descriptive Patient Characteristics and Frequency of Outcomes^a^

Characteristic	Patients, No. (%) (N = 80 669)							
	No supplemental oxygen		Supplemental oxygen		NIPPV		MV and/or ECMO	
	Control (n = 5503)	Dexamethasone (n = 7537)	Control (n = 7789)	Dexamethasone (n = 48 579)	Control (n = 792)	Dexamethasone (n = 6826)	Control (n = 1013)	Dexamethasone (n = 2660)
Age, median (IQR), y	68 (53-79)	64 (51-76)	70 (58-81)	64 (51-75)	68 (58-77)	65 (54-75)	64 (50-74)	63 (53-73)
Sex								
Female	2738 (49.8)	3641 (48.3)	4019 (51.6)	22 600 (46.5)	369 (46.6)	2744 (40.2)	420 (41.5)	1075 (40.4)
Male	2765 (50.2)	3896 (51.7)	3770 (48.4)	25 979 (53.5)	423 (53.4)	4082 (59.8)	593 (58.5)	1585 (59.6)
Race								
Black	1291 (23.5)	1610 (21.4)	1530 (19.6)	7080 (14.6)	135 (17.1)	1008 (14.8)	175 (17.3)	401 (15.1)
White	3161 (57.4)	4369 (58.0)	4901 (62.9)	30 055 (61.9)	536 (67.7)	4136 (60.6)	616 (60.8)	1448 (54.4)
Other^b^	1051 (19.1)	1558 (20.8)	1358 (17.4)	11 444 (23.6)	121 (15.3)	1682 (24.6)	222 (21.9)	811 (30.5)
Hispanic ethnicity	1238 (22.5)	1883 (25.0)	1691 (21.7)	12 710 (26.2)	149 (18.8)	1717 (25.2)	247 (24.4)	705 (26.5)
Comorbidities								
Hypertension	4270 (77.6)	5364 (71.2)	6332 (81.3)	33 845 (69.7)	679 (85.7)	5417 (79.4)	789 (77.9)	2049 (77.0)
Hyperlipidemia	2901 (52.7)	3572 (47.4)	4426 (56.8)	22 307 (45.9)	490 (61.9)	3466 (50.8)	493 (48.7)	1262 (47.4)
Chronic kidney disease	3269 (59.4)	3614 (48.0)	4930 (63.3)	24 439 (50.3)	597 (75.4)	5030 (73.7)	774 (76.4)	2190 (82.3)
Diabetes	2615 (47.5)	3120 (41.4)	3926 (50.4)	21 153 (43.5)	480 (60.6)	3855 (56.5)	537 (53.0)	1564 (58.8)
Coronary artery disease	1808 (32.9)	1974 (26.2)	2979 (38.3)	12 245 (25.2)	358 (45.2)	2655 (38.9)	429 (42.4)	1178 (44.3)
Obesity	1505 (27.4)	2386 (31.7)	2937 (37.7)	20 096 (41.4)	471 (59.5)	3987 (58.4)	440 (43.4)	1321 (49.7)
Chronic obstructive pulmonary disease	1259 (22.9)	1876 (24.9)	2704 (34.7)	12 915 (26.6)	339 (42.8)	2361 (34.6)	310 (30.6)	801 (30.1)
Charlson Comorbidity Index score								
≥2	3073 (56.7)	3197 (42.9)	4781 (62.4)	19 950 (41.8)	532 (68.5)	4003 (59.5)	658 (65.7)	1771 (67.5)
1	1296 (23.9)	2064 (27.7)	1671 (21.8)	13 532 (28.3)	147 (18.9)	1630 (24.2)	199 (19.9)	570 (21.7)
0	1054 (19.4)	2192 (29.4)	1216 (15.9)	14 268 (29.9)	98 (12.6)	1094 (16.3)	144 (14.4)	283 (10.8)
Intensive care unit admission	430 (7.8)	291 (3.86)	1211 (15.6)	5187 (10.7)	337 (42.6)	3017 (44.2)	981 (96.8)	2455 (92.3)
Ratio of oxygen saturation to fraction of inspired oxygen, median (IQR)	448 (438-452)	443 (438-452)	288 (233-329)	258 (176-314)	94 (87-184)	90 (82-126)	117 (91-192)	88 (80-98)
Medications								
Antibiotics	3940 (71.6)	6115 (81.1)	6159 (79.1)	38 855 (80.0)	666 (84.1)	6022 (88.2)	847 (83.6)	2315 (87.0)
Prophylactic anticoagulation	2720 (49.4)	3030 (40.2)	3802 (48.8)	21 943 (45.2)	402 (50.8)	2641 (38.7)	451 (44.5)	1139 (42.8)
Treatment for anticoagulation	891 (16.2)	1018 (13.5)	1712 (22.0)	9433 (19.4)	324 (40.9)	1659 (24.3)	338 (33.4)	749 (28.2)
Remdesivir	124 (2.3)	823 (12.3)	1454 (18.7)	18 830 (38.8)	317 (40.0)	2620 (38.4)	208 (20.5)	824 (31.0)
Vasopressors	15 (0.27)	5 (0.07)	66 (0.85)	125 (0.26)	15 (1.89)	60 (0.88)	263 (26.0)	530 (19.9)
Tocilizumab	0	1 (0.01)	15 (0.19)	247 (0.51)	30 (3.8)	180 (2.6)	26 (2.6)	101 (3.8)
Baricitinib	0	7 (0.09)	23 (0.30)	288 (0.59)	22 (2.8)	125 (1.8)	21 (2.1)	31 (1.2)
End points								
Mortality^c^	438 (8.0)	414 (5.5)	1357 (17.4)	6978 (14.4)	384 (48.5)	3190 (46.7)	546 (53.9)	1522 (57.2)
Mortality or hospice^d^	661 (12.0)	543 (7.2)	1748 (22.4)	8092 (16.7)	421 (53.2)	3420 (50.1)	586 (57.9)	1609 (60.5)

Abbreviations: ECMO, extracorporeal membrane oxygenation; MV, mechanical ventilation; NIPPV, noninvasive positive pressure ventilation.

^a^ Frequency of missingness for each variable is included in eTables 1 through 4 in Supplement 1.

^b^ Other race refers to Hawaiian or Pacific Islander, American Indian or Alaska Native, other, or unknown.

^c^ Refers to all-cause in-hospital mortality.

^d^ Refers to all-cause in-hospital mortality or discharge to hospice.

### Statistical Analysis

Data analysis was performed from March 2022 to February 2023. Statistical analyses were conducted in SAS statistical software version 9.4 (SAS Institute) and R statistical software version 4.2.2 (R Project for Statistical Computing). Patient and hospital characteristics were described for each cohort overall and by treatment status, with frequencies and proportions for categorical variables and medians and IQRs for continuous variables. Potential confounding by hospital-level and patient-level variables was handled by propensity score overlap weighting. Time-invariant confounders, such as age, were derived at the hospital admission date, whereas factors that may vary during hospitalization were evaluated at the cohort-specific index date, which was defined as the baseline date of eligibility in a particular cohort. The propensity score model was developed using the method of Yang et al^9^ to appropriately adjust for confounding both in the overall analytic cohorts and within a priori subgroups of interest based on age (18-50, 51-70, and ≥71 years), self-identified race (White, Black, or other [ie, Hawaiian or Pacific Islander, American Indian or Alaska Native, other, or unknown]), ethnicity (Hispanic or non-Hispanic), Charlson Comorbidity Index (CCI) score (0, 1, or ≥2), diabetes, receipt of remdesivir, and maximum daily supplemental oxygen flow (low flow or high flow). Race and ethnicity were evaluated in this study because of the racial and ethnic disparaties associated with outcomes among patients with COVID-19. In short, this method includes both confounders, a priori subgroups and the pairwise interactions between these in the candidate logistic regression propensity score model, and then uses least absolute shrinkage and selection operator to select only the important pairwise interactions.^9^ The propensity scores are obtained as the estimated probability of receiving dexamethasone from this model and are converted into propensity score overlap weights.^9,10,11^ The validity of this approach is evaluated in a connect-S plot that shows standardized differences, overall and within prespecified subgroups, after applying the overlap weights. Standardized differences less than 0.10 indicate good balance. When good balance could not be achieved for small subgroups within a particular cohort, adjacent subgroup levels were collapsed for that cohort. For comparison, we evaluated balance diagnostics through the connect-S plot using the more common approach of inverse probability of treatment weighting based on a main effects logistic regression propensity score model (eFigures 5-16 in Supplement 1).^9^ Good balance was not achieved by this method, and it was not used for analysis of outcomes. The association between dexamethasone treatment and in-hospital mortality was assessed through logistic regression with adjustment via overlap weighting. Odds ratios (ORs) are reported overall and within subgroups; 95% CIs were estimated by the robust sandwich estimator to account for estimation of the propensity score weights (eFigures 17-20 in Supplement 1). Hypothesis testing was not a primary goal of this research, and 95% CIs rather than *P* values are emphasized. However, we include 2-sided *P* values for the test of interaction between treatment effects and subgroups of interest, to formalize the comparisons and recognize chance variability, with *P* < .05 denoting significance. These are nominal *P* values without adjustment for multiple testing.

#### Handling of Missing Data

Missing data were not imputed for univariable descriptive tables, and rates of missingness are reported in eTables 1 through 4 in Supplement 1. The handling of missing data is described in eAppendix 3 and eTable 5 in Supplement 1. In short, intermittent missingness for variables measured daily during the hospital was addressed through last value carried forward, and time-invariant variables were addressed through imputation by the full-conditional specific method in SAS PROC MI. Missing data were rare for time-invariant variables, and the results of single and multiple imputation were nearly identical (eAppendix 3 and eTable 5 in Supplement 1).

#### Sensitivity Analysis

Sensitivity analysis was conducted to evaluate the potential impact of time epochs in which the variant and vaccination status changed. We prespecified 2 periods of interest: before and after January 1, 2021. We selected these periods because the former period preceded widespread vaccination and predominantly included the SARS-CoV-2 Alpha variant, whereas the latter period predominantly included the SARS-CoV-2 Delta variant and vaccination was more common, making these populations theoretically distinct.

## Results

### Patient Characteristics

After applying population-level and cohort-level inclusion and exclusion criteria (eFigures 3 and 4 in Supplement 1), 80 699 patients met the eligibility criteria (median [IQR] age, 64 [52-76] years; 37 606 women [46.6%]); 13 230 patients (16.4%) identified as Black, 49 222 (60.9%) as White, 18 247 (22.6%) as other race, and 20 340 (25.2%) as Hispanic ethnicity (Table 1). Our 4 cohorts included 13 040 (16.2%) patients who did not require supplemental oxygen or respiratory support, 56 368 patients (69.8%) who required supplemental oxygen, 7618 patients (9.4%) who required NIPPV, and 3673 patients (4.6%) who required MV and/or ECMO (eTables 1-4 in Supplement 1). Notably, cohorts receiving higher levels of oxygen support had greater proportions of patients with 2 or more comorbidities. A total of 50 908 patients (90.3%) in our supplemental oxygen group required low-flow supplemental oxygen. A total of 25 200 patients (31.2%) were treated with remdesivir before initiating dexamethasone as specified, although this was much lower in the cohort without supplemental oxygen. The median (IQR) duration of therapy with dexamethasone ranged from 4 (3-6) days to 9 (5-11) days, and the median (IQR) daily dose ranged from 7.3 (6.0-12.0) mg to 11.0 (6.1-12.0) mg across groups (Table 2). Among controls who did not receive early dexamethasone, 1655 (30.1%) in the no supplemental oxygen cohort, 3583 (46.0%) in the supplemental oxygen cohort, 283 (35.7%) in the NIPPV cohort, and 299 (29.5%) in the MV and/or ECMO cohort later went on to receive dexamethasone during their admission.

**Table 2.** Characteristics of Treatment Among Dexamethasone and Control Groups

Treatment group	Median (IQR)			
	No supplemental oxygen	Supplemental oxygen	NIPPV	MV and/or ECMO
Dexamethasone				
Duration of therapy, d	4 (3-6)	6 (4-9)	9 (5-11)	9 (5-10)
Total dose, mg^a^	34 (18-54)	48 (30-80)	78 (46-132)	72 (42-120)
Daily dose, mg	7.3 (6.0-12.0)	7.5 (6.0-12.0)	10.8 (6.6-13.2)	11.0 (6.1-12.0)
Control, received dexamethasone, No. (%), patients^b^	1655 (30.1)	3583 (46.0)	283 (35.7)	299 (29.5)

Abbreviations: ECMO, extracorporeal membrane oxygenation; MV, mechanical ventilation; NIPPV, noninvasive positive pressure ventilation.

^a^ Refers to total milligrams of dexamethasone received during hospitalization.

^b^ Refers to patients who received dexamethasone after 48 hours of admission or escalation in oxygen support.

### Primary Outcome

Descriptive outcomes and unadjusted ORs are shown in eTable 6 and eTable 7 in Supplement 1. Propensity score overlap weighting was successful in creating weighted populations with comparable characteristics both overall and within subgroups (eFigures 5-16 in Supplement 1). After overlap weighting, all-cause inpatient mortality or discharge to hospice was lower for patients who received dexamethasone within 48 hours of either admission or escalation in oxygen support in the supplemental oxygen group (8092 patients [16.7%] vs 1748 patients [22.4%]; adjusted OR [aOR], 0.92; 95% CI, 0.86-0.98) (Table 1 and Figure, panel B) and in the MV and/or ECMO group (1609 patients [60.5%] vs 586 patients [57.9%]; aOR, 0.82; 95% CI, 0.68-0.99) (Figure, panel D). In contrast, all-cause inpatient mortality or discharge to hospice was not lower for patients who received dexamethasone in the no supplemental oxygen group (543 patients [7.2%] vs 661 patients [12.0%]; aOR, 0.90; 95% CI, 0.78-1.03) (Figure, panel A) and in the NIPPV group (3420 patients [50.1%] vs 421 patients [53.2%]; aOR, 0.87; 95% CI, 0.73-1.04) (Figure, panel C).

**Figure.**
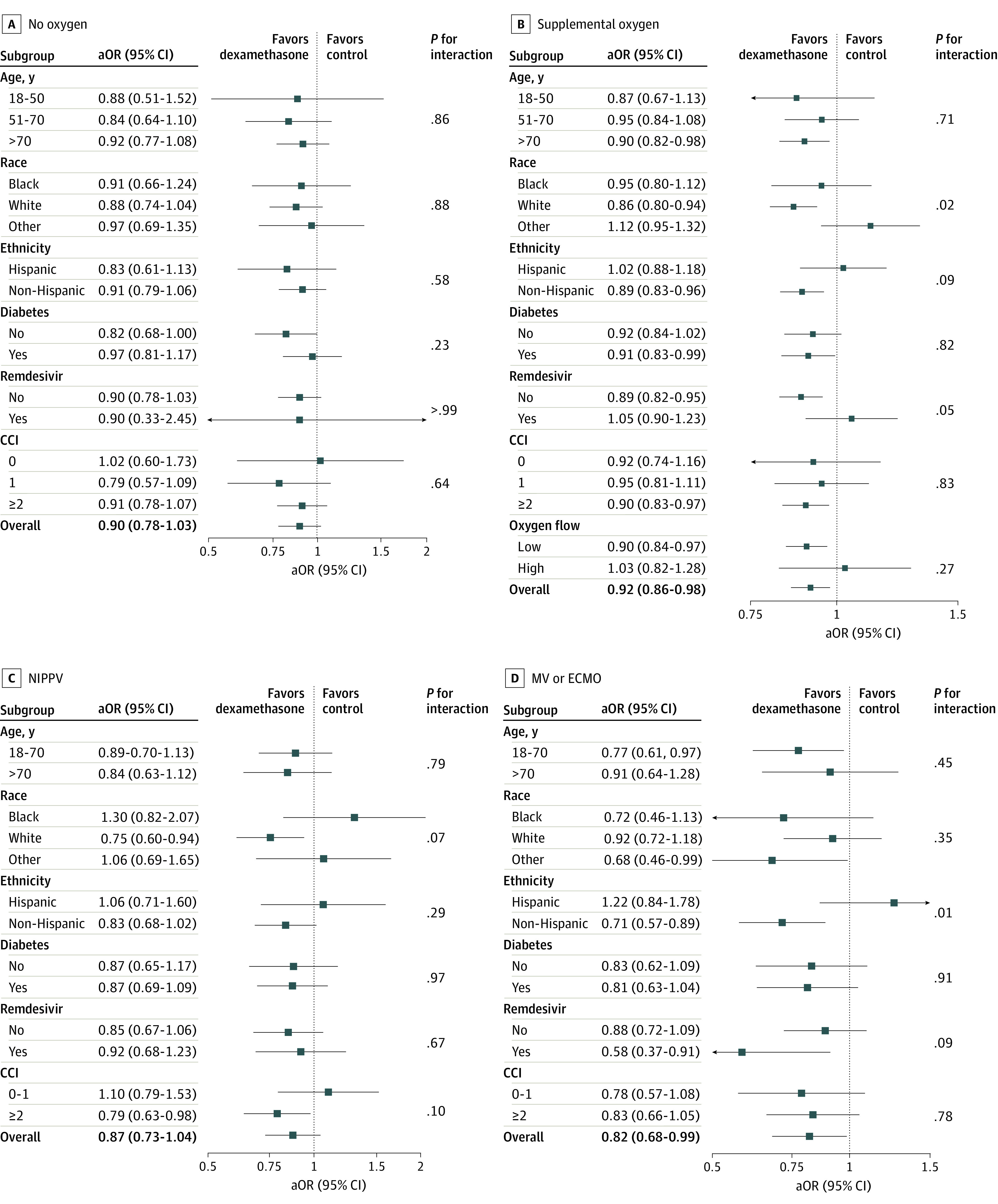
Analysis of Mortality or Discharge to Hospice Forest plots show adjusted odds ratios (aORs) for patients not receiving supplemental oxygen (A), patients receiving supplemental oxygen (B), patients receiving noninvasive positive pressure ventilation (NIPPV) (C), and patients receiving mechanical ventilation (MV) and/or extracorporeal membrane oxygenation (ECMO) (D). Other race refers to Hawaiian or Pacific Islander, American Indian or Alaska Native, other, or unknown. CCI indicates Charlson Comorbidity Index score.

### Subgroup Analyses

In prespecified subgroup analysis among patients receiving supplemental oxygen (Figure, panel B), improvement in the primary outcome was seen in patients older than 70 years (aOR, 0.90; 95% CI, 0.82-0.98), White patients (aOR, 0.86; 95% CI, 0.80-0.94), non-Hispanic patients (aOR, 0.89; 95% CI, 0.83-0.96), patients with diabetes (aOR, 0.91; 95% CI, 0.83-0.99), patients who did not receive remdesivir (aOR, 0.89; 95% CI, 0.82-0.95), patients with higher CCI score (aOR, 0.90; 95% CI, 0.83-0.97), and patients receiving low-flow supplemental oxygen (aOR, 0.90; 95% CI, 0.84-0.97). Among patients requiring NIPPV (Figure, panel C), improvement in the primary outcome was seen in White patients (aOR, 0.75; 95% CI, 0.60-0.94) and patients with higher CCI score (aOR, 0.79; 95% CI, 0.63-0.98). Finally, among patients requiring MV and/or ECMO (Figure, panel D), improvement in the primary outcome was specifically seen in younger patients (aOR, 0.77; 95% CI, 0.61-0.97), non-Hispanic patients (aOR, 0.71; 95% CI, 0.57-0.89), and patients who received remdesivir (aOR, 0.58; 95% CI, 0.37-0.91). In sensitivity analyses, the results show consistency in the association between dexamethasone and outcome by time epoch (before vs after January 1, 2021), as well as consistency in the results for other subgroups within our primary analysis (eFigures 21-24 in Supplement 1).

## Discussion

In this large propensity score–adjusted cohort analysis of hospitalized patients with COVID-19 respiratory illness who received dexamethasone within 48 hours of either admission or escalation in oxygen support, we identified 3 key observations. First, the administration of dexamethasone on or within 2 days of hospital admission or escalation in oxygen support improved all-cause inpatient mortality or discharge to hospice among patients receiving supplemental oxygen or MV and/or ECMO. Second, the results were directionally consistent regardless of oxygen support; however, there was no benefit or harm observed with dexamethasone use in hospitalized patients not receiving supplemental oxygen or NIPPV. Third, in a large US cohort, with overall lower mortality than seen in the RECOVERY trial,^2^ dexamethasone still proved beneficial in hospitalized patients requiring respiratory support. This analysis provides additional insight into the use of dexamethasone in specific patient subpopulations; notably, its use was associated with improved outcomes in older patients and in those with more comorbidities.

Given the heterogeneity of acute respiratory distress syndrome, including causes and mechanisms of respiratory failure, the utility of and indications for corticosteroid therapy for acute respiratory distress syndrome are highly variable.^12,13^ Several studies^14,15,16^ have assessed the use of corticosteroids in patients with respiratory failure specifically of viral causes before the COVID-19 pandemic, including influenza, Middle East respiratory syndrome, and other coronaviruses, with some studies suggesting no benefit or an association with increased mortality. The RECOVERY trial^2^ and subsequent studies assessing the use of dexamethasone for COVID-19 pneumonia and respiratory failure have greatly reshaped clinical practice. Our data build on these studies and fill in knowledge gaps regarding the use of dexamethasone in key patient subgroups.

Preexisting comorbidities are common in patients hospitalized with COVID-19, and those with preexisting comorbidities are more likely to have severe COVID-19, as well as worse outcomes from their illness.^17,18,19,20,21^ In our cohort, the distribution of preexisting comorbidities was consistent with previously published trends,^19,20,21^ and patients with higher CCI score in the supplemental oxygen and NIPPV groups had the greatest benefit from dexamethasone. This reflects the heterogeneity of effect of corticosteroids. Although we did not observe harm from administering dexamethasone in younger, healthier patients, these results suggest that respiratory status alone does not determine the efficacy of dexamethasone and that patients who are more likely to have worse outcomes from COVID-19 may also be the patients who benefit most from therapy.

Although the RECOVERY study^2^ assessed dexamethasone use in patients requiring different levels of respiratory support, the group requiring supplemental oxygen was not explored in more detail by level of supplemental oxygen flow required or the need for NIPPV. In our cohort, we were able to stratify patients into those requiring low-flow and high-flow supplemental oxygen, as well as those receiving NIPPV. Approximately 90% of patients in our supplemental oxygen group required low-flow supplemental oxygen, and the greatest benefit with dexamethasone was among these patients. Although there was a benefit for patients receiving high-flow supplemental oxygen, our analysis of this subgroup was limited by the small sample size relative to the other subgroups. Interestingly, we did not see a benefit to dexamethasone use in patients requiring NIPPV despite there being benefit in both the supplemental oxygen and MV and/or ECMO groups. The majority of patients in the NIPPV cohort received early dexamethasone, and 5820 controls switched during follow-up, yielding a very small number of controls. Thus, the 95% CI is wide and includes the possibility of no association, as well as benefit that is consistent with the adjacent oxygen cohorts. One explanation for the lack of benefit observed in this group is that we could not account for baseline NIPPV use (ie, patients with obstructive sleep apnea or structural lung disease requiring continuous positive airway pressure or bilevel positive airway pressure at baseline that were resumed during hospitalization), which may have resulted in an overestimate the degree of respiratory distress. Dexamethasone use in patients receiving NIPPV was not explored in most clinical trials or observational studies, and our results suggest there is more to be learned about this population.

Several studies^22,23^ have shown differences in the effects of antiviral medications, such as remdesivir, on outcomes in patients with COVID-19 on the basis of the level of respiratory support they are receiving, with most recent results showing no improvement in mortality in those requiring mechanical ventilation. Although our study was not designed to assess the effect of remdesivir on the primary outcome, patients receiving MV and/or ECMO who received remdesivir saw greater benefit with dexamethasone. In addition, patients receiving supplemental oxygen who were not receiving remdesivir saw benefit with dexamethasone. Although these differences may be partially attributable to the effects of remdesivir, they may also reflect patterns of care and the distinct characteristics of patients who do or do not receive remdesivir in these 2 cohorts.

Throughout the pandemic there has been a pattern of racial, ethnic, and socioeconomic disparities associated with outcomes of patients with COVID-19.^24^ In our cohort we saw similar patterns. White and non-Hispanic patients who received supplemental oxygen had improved mortality with dexamethasone, with similar trends in the NIPPV group. For Black and Hispanic patients, the 95% CIs were wide and difficult to interpret. More research is needed to understand potential causes for these differences.

Among patients not requiring supplemental oxygen, studies have shown harm associated with administration of dexamethasone.^2,25^ The reason for this is still unknown but is hypothesized to be caused by early administration of corticosteroids before respiratory distress, resulting in complications from adverse systemic effects of the corticosteroids. Although we did not see benefit in administering dexamethasone to these patients in our cohort, we also did not see any harm. There may have been other indications for administration of corticosteroids or uncaptured confounders in these patients that account for this pattern. What is striking, however, is that these patients with no guideline-based indication for corticosteroid use for COVID-19 pneumonia received early therapy. This speaks to the variability in clinical practice despite published clinical trials and society guidelines.

### Limitations

Although our study was conducted on a large, national, multicenter cohort, with robust statistical modeling to minimize bias and explore patient subgroups in more detail, it does have several limitations. We did not have data on vaccination status or days since symptom onset, both of which could have affected the treatment response and our interpretation of several administered medications, including antivirals and corticosteroids. Furthermore, given that circulating SARS-CoV-2 variant strains and standard of care for both inpatient and outpatient management and prevention of COVID-19, including medication administration and vaccination, changed throughout the study period,^26,27,28^ patient outcomes in our cohort varied over time, which could have affected our measured effect size, as well as the comparability of propensity-weighted data within this study period. In addition, dexamethasone was used so widely in this cohort that we had few control patients, and many of them later went on to receive corticosteroids during their hospital stay. This led to wide 95% CIs for some subgroups and potentially low power for the tests of interaction. The interpretation of our result is applicable to a setting with similar levels of crossover, which could diminish the strength of association with dexamethasone compared with the effect that would be seen if patients adhered to an initial treatment group assignment. *P* values were not adjusted for multiple testing; therefore, caution is required considering multiple cohorts and multiple subgroups. Furthermore, despite using propensity score overlap weighting, unmeasured confounders may have influenced the results, and balance of the measured covariates was not always perfect.

## Conclusions

In our large national cohort study of patients with COVID-19 respiratory illness hospitalized between July 1, 2020, and October 31, 2021, early administration of dexamethasone was associated with improved mortality or discharge to hospice in those requiring supplemental oxygen or MV and/or ECMO. Continued exploration of patient subgroups will help inform and individualize therapy for COVID-19. Overall, these results demonstrate that despite the evolution of the COVID-19 pandemic over time, dexamethasone remains beneficial for these hospitalized patients in a clinical practice setting.

## Notes

Supplement 1. **eFigure 1.** HCA Healthcare Geographic Representation **eFigure 2.** HCA Starting Population Algorithm **eAppendix 1.** Variables Identified as Confounders and Used for Adjustment **eAppendix 2.** Study-Specific Inclusion and Exclusion Criteria **eFigure 3.** Consort Diagram of Exclusions Applied to the Starting Population **eFigure 4.** Cohort-Specific Selection Applied at the Time of Eligibility for Each **eTable 1. **Characteristics of Patients Eligible for the No Supplemental Oxygen Cohort **eTable 2.** Characteristics of Patients Eligible for the Supplemental Oxygen Cohort **eTable 3.** Characteristics of Patients Eligible for the Noninvasive Positive Pressure Ventilation (NIPPV) Cohort **eTable 4.** Characteristics of Patients Eligible for the Mechanical Ventilation (MV) or Extracorporeal Membrane Oxygenation (ECMO) Cohort **eFigure 5.** Unadjusted Connect-S Plot for No Supplemental Oxygen Cohort **eFigure 6.** IPW-Logistic Connect-S Plot for No Supplemental Oxygen Cohort **eFigure 7.** OW Adjusted Connect-S Plot for No Supplemental Oxygen Cohort **eFigure 8.** Unadjusted Connect-S Plot for Supplemental Oxygen Cohort **eFigure 9. **IPW-Logistic Connect-S Plot for Supplemental Oxygen Cohort **eFigure 10. **OW Adjusted Connect-S Plot for Supplemental Oxygen Cohort **eFigure 11.** Unadjusted Connect-S Plot for NIPPV Cohort **eFigure 12.** IPW-Logistic Connect-S Plot for NIPPV Cohort **eFigure 13.** OW Adjusted Connect-S Plot for NIPPV Cohort **eFigure 14.** Unadjusted Connect-S Plot for MV or ECMO Cohort **eFigure 15.** IPW-Logistic Connect-S Plot for MV or ECMO Cohort **eFigure 16.** OW Adjusted Connect-S Plot for MV or ECMO Cohort **eFigure 17.** Histogram of the Propensity Score Distribution Overlaid by Treatment After Overlap Weighting for No Supplemental Oxygen Cohort **eFigure 18.** Histogram of the Propensity Score Distribution Overlaid by Treatment After Overlap Weighting for Supplemental Oxygen Cohort **eFigure 19.** Histogram of the Propensity Score Distribution Overlaid by Treatment After Overlap Weighting for NIPPV Cohort **eFigure 20.** Histogram of the Propensity Score Distribution Overlaid by Treatment After Overlap Weighting for MV or ECMO Cohort **eAppendix 3.** Handling of Missing Data and Multiple Imputation **eTable 5.** Adjusted Odds Ratios of Single Imputed Data Set and Multiply Imputed Data Set **eTable 6.** Frequency of End Points per Cohort **eTable 7.** Unadjusted Analysis of In-Hospital Mortality or Discharge to Hospice **eFigure 21.** Time Epoch Sensitivity Analysis for Patients on No Supplemental Oxygen **eFigure 22.** Time Epoch Sensitivity Analysis for Patients on Supplemental Oxygen **eFigure 23.** Time Epoch Sensitivity Analysis for Patients on NIPPV **eFigure 24.** Time Epoch Sensitivity Analysis for Patients on MV/ECMO

Supplement 2.
Data Sharing Statement
