# Supplementary material for: Dexamethasone for Inpatients With COVID-19 in a National Cohort
Source: JAMA Netw Open. 2023 Apr 17;6(4):e238516. doi: 10.1001/jamanetworkopen.2023.8516 (PMC10111178; doi:10.1001/jamanetworkopen.2023.8516)
Supplement: Supplement 1. — eFigure 1. HCA Healthcare Geographic Representation eFigure 2. HCA Starting Population Algorithm eAppendix 1. Variables Identified as Confounders and Used for Adjustment eAppendix 2. Study-Specific Inclusion and Exclusion Criteria eFigure 3. Consort Diagram of Exclusions Applied to the Starting Population eFigure 4. Cohort-Specific Selection Applied at the Time of Eligibility for Each eTable 1. Characteristics of Patients Eligible for the No Supplemental Oxygen Cohort eTable 2. Characteristics of Patients Eligible for the Supplemental Oxygen Cohort eTable 3. Characteristics of Patients Eligible for the Noninvasive Positive Pressure Ventilation (NIPPV) Cohort eTable 4. Characteristics of Patients Eligible for the Mechanical Ventilation (MV) or Extracorporeal Membrane Oxygenation (ECMO) Cohort eFigure 5. Unadjusted Connect-S Plot for No Supplemental Oxygen Cohort eFigure 6. IPW-Logistic Connect-S Plot for No Supplemental Oxygen Cohort eFigure 7. OW Adjusted Connect-S Plot for No Supplemental Oxygen Cohort eFigure 8. Unadjusted Connect-S Plot for Supplemental Oxygen Cohort eFigure 9. IPW-Logistic Connect-S Plot for Supplemental Oxygen Cohort eFigure 10. OW Adjusted Connect-S Plot for Supplemental Oxygen Cohort eFigure 11. Unadjusted Connect-S Plot for NIPPV Cohort eFigure 12. IPW-Logistic Connect-S Plot for NIPPV Cohort eFigure 13. OW Adjusted Connect-S Plot for NIPPV Cohort eFigure 14. Unadjusted Connect-S Plot for MV or ECMO Cohort eFigure 15. IPW-Logistic Connect-S Plot for MV or ECMO Cohort eFigure 16. OW Adjusted Connect-S Plot for MV or ECMO Cohort eFigure 17. Histogram of the Propensity Score Distribution Overlaid by Treatment After Overlap Weighting for No Supplemental Oxygen Cohort eFigure 18. Histogram of the Propensity Score Distribution Overlaid by Treatment After Overlap Weighting for Supplemental Oxygen Cohort eFigure 19. Histogram of the Propensity Score Distribution Overlaid by Treatment After Overlap Weighting for NIPPV Cohort eFigure 20. Histogram of [file jamanetwopen-e238516-s001.pdf]

Supplemental Online Content

Mourad A, Thibault D, Holland TL, et al. Dexamethasone for inpatients with COVID-19 in a national cohort. *JAMA Netw Open*. 2023;6(4):e238516. doi:10.1001/jamanetworkopen.2023.8516

eFigure 1. HCA Healthcare Geographic Representation

eFigure 2. HCA Starting Population Algorithm

eAppendix 1. Variables Identified as Confounders and Used for Adjustment

eAppendix 2. Study-Specific Inclusion and Exclusion Criteria

eFigure 3. Consort Diagram of Exclusions Applied to the Starting Population

eFigure 4. Cohort-Specific Selection Applied at the Time of Eligibility for Each

eTable 1. Characteristics of Patients Eligible for the No Supplemental Oxygen Cohort

eTable 2. Characteristics of Patients Eligible for the Supplemental Oxygen Cohort

eTable 3. Characteristics of Patients Eligible for the Noninvasive Positive Pressure Ventilation (NIPPV) Cohort

eTable 4. Characteristics of Patients Eligible for the Mechanical Ventilation (MV) or Extracorporeal Membrane Oxygenation (ECMO) Cohort

eFigure 5. Unadjusted Connect-S Plot for No Supplemental Oxygen Cohort

eFigure 6. IPW-Logistic Connect-S Plot for No Supplemental Oxygen Cohort

eFigure 7. OW Adjusted Connect-S Plot for No Supplemental Oxygen Cohort

eFigure 8. Unadjusted Connect-S Plot for Supplemental Oxygen Cohort

eFigure 9. IPW-Logistic Connect-S Plot for Supplemental Oxygen Cohort

eFigure 10. OW Adjusted Connect-S Plot for Supplemental Oxygen Cohort

eFigure 11. Unadjusted Connect-S Plot for NIPPV Cohort

eFigure 12. IPW-Logistic Connect-S Plot for NIPPV Cohort

eFigure 13. OW Adjusted Connect-S Plot for NIPPV Cohort

eFigure 14. Unadjusted Connect-S Plot for MV or ECMO Cohort

eFigure 15. IPW-Logistic Connect-S Plot for MV or ECMO Cohort

eFigure 16. OW Adjusted Connect-S Plot for MV or ECMO Cohort

© 2023 Mourad A et al. *JAMA Network Open.*

eFigure 17. Histogram of the Propensity Score Distribution Overlaid by Treatment After Overlap Weighting for No Supplemental Oxygen Cohort

eFigure 18. Histogram of the Propensity Score Distribution Overlaid by Treatment After Overlap Weighting for Supplemental Oxygen Cohort

eFigure 19. Histogram of the Propensity Score Distribution Overlaid by Treatment After Overlap Weighting for NIPPV Cohort

eFigure 20. Histogram of the Propensity Score Distribution Overlaid by Treatment After Overlap Weighting for MV or ECMO Cohort

eAppendix 3. Handling of Missing Data and Multiple Imputation

eTable 5. Adjusted Odds Ratios of Single Imputed Data Set and Multiply Imputed Data Set

eTable 6. Frequency of End Points per Cohort

eTable 7. Unadjusted Analysis of In-Hospital Mortality or Discharge to Hospice

eFigure 21. Time Epoch Sensitivity Analysis for Oatients on No Supplemental Oxygen

eFigure 22. Time Epoch Sensitivity Analysis for Patients on Supplemental Oxygen

eFigure 23. Time Epoch Sensitivity Analysis for Patients on NIPPV

eFigure 24. Time Epoch Sensitivity Analysis for Patients on MV/ECMO

This supplemental material has been provided by the authors to give readers additional information about their work.

© 2023 Mourad A et al. *JAMA Network Open.*

eFigure 1. HCA Healthcare Geographic Representation

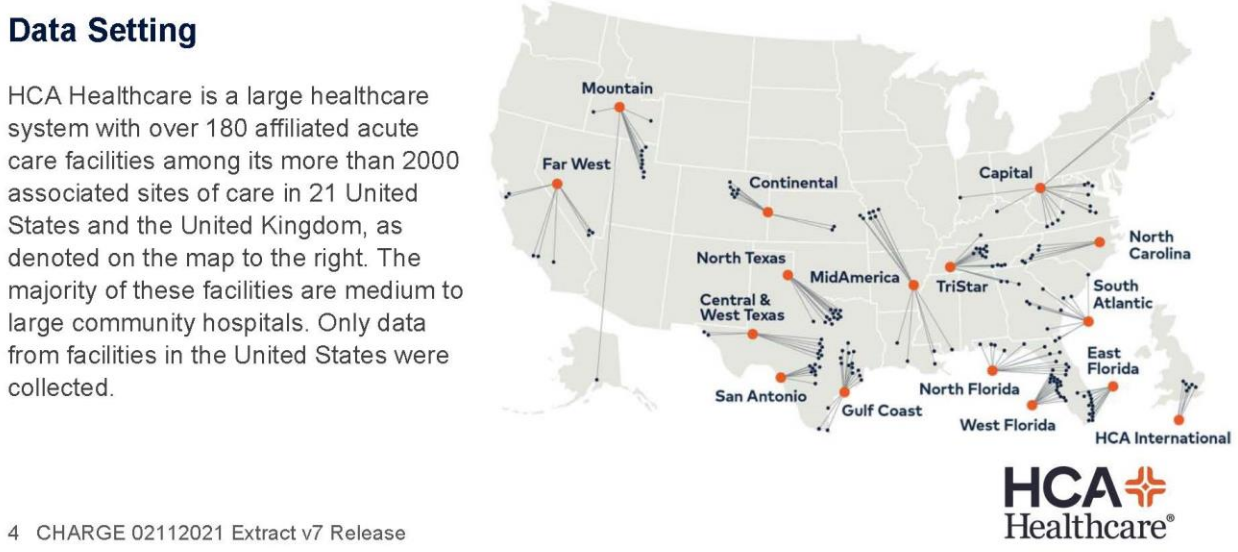

eFigure 2. HCA Starting Population Algorithm

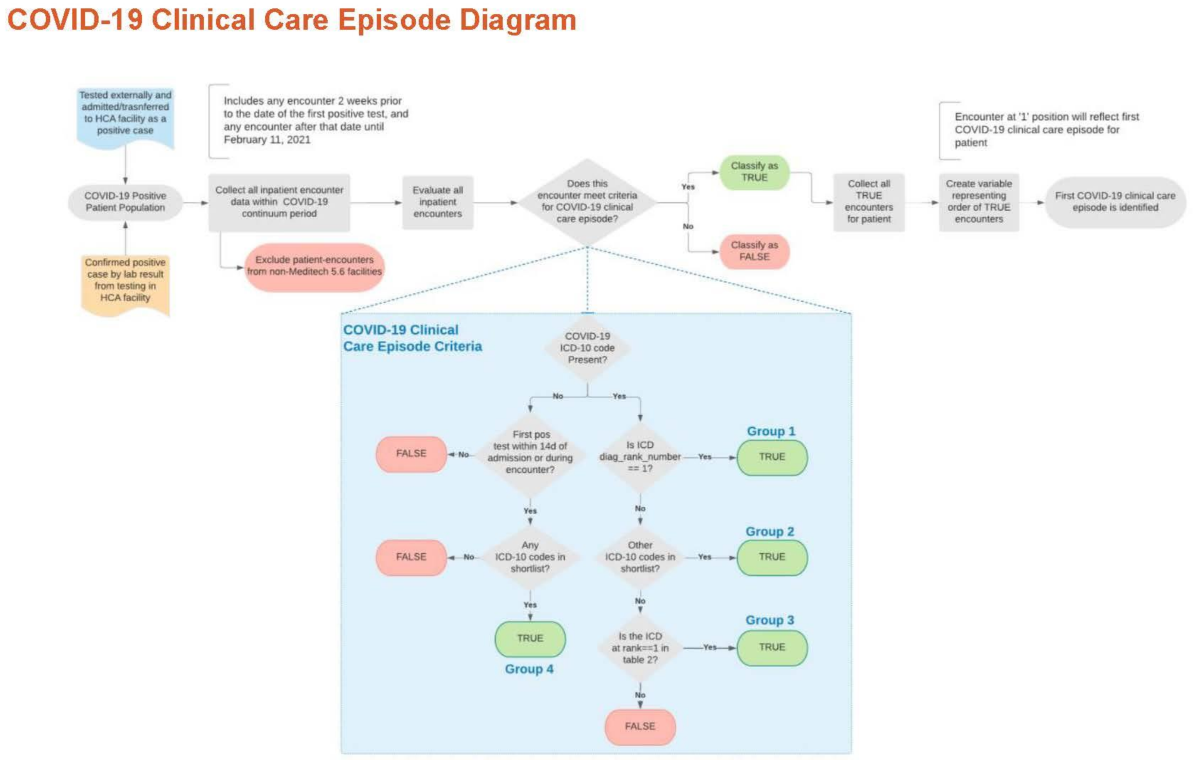

© 2023 Mourad A et al. *JAMA Network Open.*

eAppendix 1. Variables Identified as Confounders and Used for Adjustment

The list of potential confounding variables was identified through Stakeholder input and consultation with the HCA Healthcare / CHARGE Consortium Steering Committee.

Our causal model to define confounders was to enumerate all factors thought to be common causes of dexamethasone initiation and outcome, with particular emphasis on those factors that precede treatment and might be strongly related to outcome, as these are known to improve finite-sample efficiency, even if they don't play an explicit role in treatment decisions. From a clinical perspective, the question was: What factors might play a role in deciding whether to initiate dexamethasone that also impact mortality in the setting of COVID-19? Our team developed a list of such variables based on the literature and shared it with the HCA Healthcare / CHARGE Consortium Steering Committee.

The resulting list is as follows:

Community/Hospital level variables:

- Hospital volume (bed size)
- Hospital ICU volume (ICU bed size)
- Region (4 category variable)

Patient variables:

- Calendar time (measured continuously to capture changes over time)
- Time epoch: (Note Epochs are defined based on prior HCA research and periods 1-4 do not occur in our population)
  - 1="Epoch 1: March 2 - April 2, 2020"
  - 2="Epoch 2: April 3 - April 29, 2020"
  - 3="Epoch 3: April 30 - May 19, 2020"
  - 4="Epoch 4: May 20 - July 5, 2020"
  - 5="Epoch 5: July 6 - August 23, 2020"
  - 6="Epoch 6: August 24 - December 31, 2020"
  - 7="Epoch 7: Q1 2021"
  - 8="Epoch 8: Q2 2021"
  - 9="Epoch 9: Q3 2021"
  - 10="Epoch 10: Q4 2021"
- Age
- Sex
- Race/ethnicity (African American, Caucasian, Hispanic, others: American Indians, Asians) (race / ethnicity)
- Smoking
  - Smoking status categories:
    - Current smoker or smoker, current status unknown

© 2023 Mourad A et al. *JAMA Network Open.*

    - Former smoker
    - Never smoker
    - Unknown
- Minimum Spo2 at baseline
- BMI
  - BMI is derived from height and weight. Prior to calculating BMI we cleaned up height and weight using the following logic
    - Weight: values <50 lbs or >1000 lbs were set to missing
    - Height: values <3 ft or >8 ft were set to missing

- Medical history

  For the following conditions we only used historical data (i.e. information from diagnosis history dataset which includes historical diagnoses captured in the EHR for the included patient population from January 1st 2018 to date, as well as any encounter that occurred before their index COVID encounter):
  - Prior stroke
  - Prior revascularization
  - Prior myocardial infarction
  - Prior pulmonary embolism
  - Charlson comorbidity index

  Historical procedures only:
  - Prior dialysis

  The conditions below could occur historically as defined above on page 4 or also on their index COVID encounter:
  - Hypertension
  - Hyperlipidemia
  - Coronary artery disease (CAD)
  - Diabetes
  - Pre-diabetes
  - Obesity
  - Autoimmune disease
  - Structural lung disease defined as a composite of:
    - Asthma
    - Chronic obstructive pulmonary disease (COPD)
    - Sarcoidosis
    - Interstitial lung disease (ILD/IPF)
  - HIV/AIDS
  - Cancer including the following separately:
    - Lymphoma / Leukemia
    - Solid tumors / malignancy
  - Chronic Kidney Disease (CKD)

Factors that may vary over the hospitalization. These were evaluated at their most recent value upon eligibility for a particular cohort.

© 2023 Mourad A et al. *JAMA Network Open.*

- Vital signs
    - Daily minimum oxygen saturation (SpO2)
    - SPO2/FiO2 ratio. SpO2 to FiO2 ratio was calculated for each patient using the lowest SpO2 and highest FiO2 of the day.
        - Fraction of inspired oxygen (FiO2) was frequently missing and handled as follows. For those with missing FiO2 in the no-oxygen group then missing was imputed to 21%. For those with missing FiO2 in the oxygen group, liters per minute of oxygen (LPM) was used to impute FiO2 by the following formula: 1 L/min = 24% FiO2, and 4% FiO2 for every 1 L/min added to that (Duprez F, et al. A New Formula for Predicting the Fraction of Delivered Oxygen During Low-Flow Oxygen Therapy. Respir Care. 2018 Dec;63(12):1528-1534. doi: 10.4187/respcare.06243. PMID: 30467224)
- Hospital COVID prevalence
    - Daily COVID numbers at the hospital divided by the hospital bed size (number of patients relative to hospital capacity) averaged over the past 7 days at the hospital
- Labs
    - Creatinine converted to eGFR (Cockroft Gault)
- In hospital interventions
    - Time since baseline (since admission date of COVID-19 hospitalizations until eligibility for a particular cohort)
    - Intensive care unit (ICU) Status (daily binary variable: 1 if in the ICU and 0 otherwise, based on icu_start_date, icu_stop_date)
    - Modified WHO ordinal scale: used to define cohorts (effectively non-parametric adjustment)
        - 2 = no supplemental oxygen received
        - 3 = received low-flow supplemental oxygen
        - 4 = received non-invasive or high-flow oxygen (CPAP, BIPAP)
        - 5 = received invasive mechanical ventilation
        - 6 = ECMO
- In hospital medications
    - Anti-coagulation (Heparin or Enoxaparin)
        - Prophylactic use
        - Treatment use
    - Remdesivir
    - Pressers (Norepinephrine, Epinephrine, Vasopressin, Phenylephrine, Dopamine)
    - Tociluzimab (in baseline table – too rare for modeling)
    - Baricitinib
    - Bamlanivimab + Etesevimab (in baseline table – too rare for modeling)
    - Casirivimab + Imdevimab (in baseline table – too rare for modeling)
    - Antibiotics
    - Azithromycin

© 2023 Mourad A et al. *JAMA Network Open.*

eAppendix 2. Study-Specific Inclusion and Exclusion Criteria

Starting population: All adults (age >17) hospitalized alive for at least 48 hours, between July 1, 2020 - October 31st 2021 for COVID-19 per CHARGE Consortium algorithm (if multiple hospitalizations meet the criteria this algorithm takes first as the index hospitalization) and having ICD-10 DX code for COVID-19 (in any position) AND ICD-10 DX code for respiratory illnesses (defined below)*

*ICD-10 DX Codes (in any position or rank, and A/I or F):

- COVID-19
  - U07.1,
- Acute Respiratory Illness
  - **J12.82 Pneumonia due to coronavirus disease 2019**
  - J12.89, Other viral pneumonia
  - J20.8, Acute Bronchitis due to other specified organisms
  - J40, Bronchitis not specified as acute or chronic
  - J22, Unspecified acute lower respiratory infection
  - J98.8, Other specified respiratory disorders
  - J80, Acute Respiratory Distress Syndrome
  - J96.00-J96.02, Acute Respiratory Failure
  - J96.2, Acute and chronic respiratory failure

© 2023 Mourad A et al. *JAMA Network Open.*

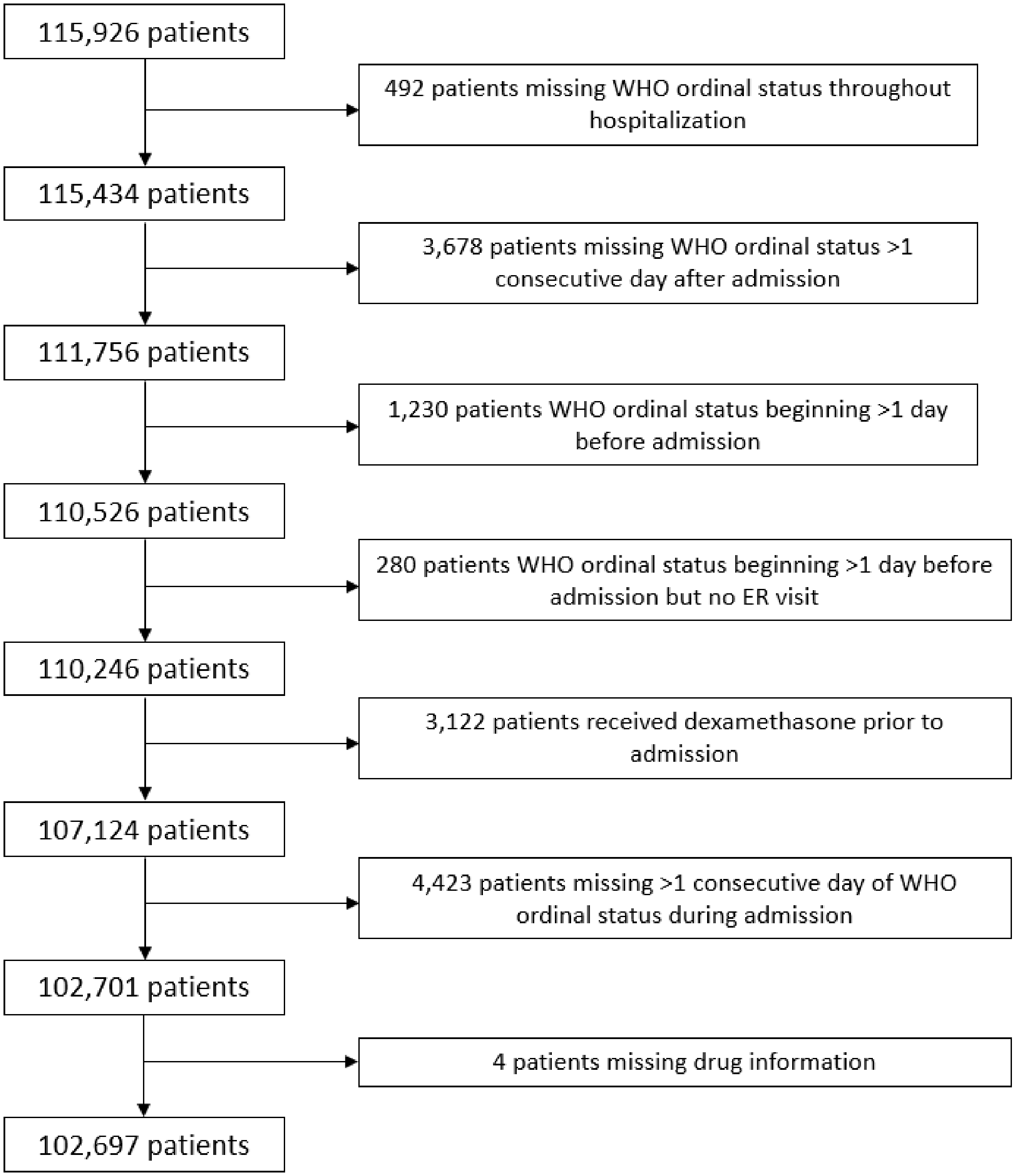

eFigure 3. Consort Diagram of Exclusions Applied to the Starting Population

© 2023 Mourad A et al. *JAMA Network Open.*

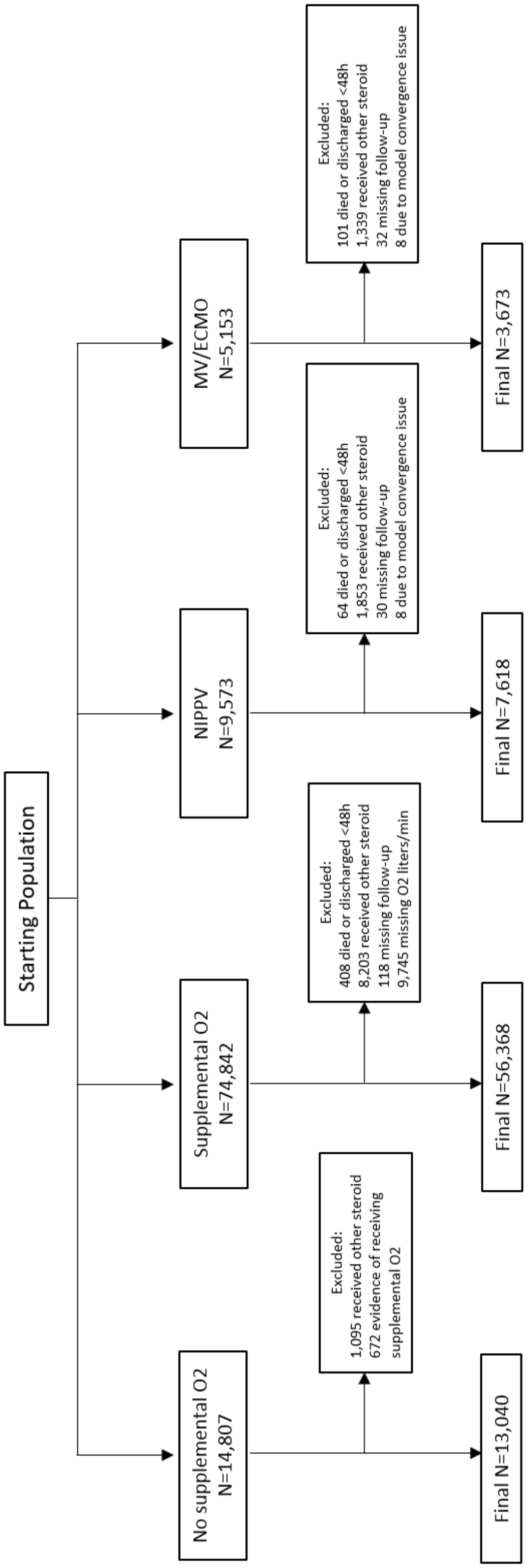

eFigure 4. Cohort-Specific Selection Applied at the Time of Eligibility for Each Cohort

© 2023 Mourad A et al. *JAMA Network Open.*

eTable 1. Characteristics of Patients Eligible for the No Supplemental Oxygen Cohort

Variable	Level	Overall (N=13040)		Control (N=5503)		Dexamethasone (N=7537)		Std Diff Score*
**Demographics**								
Hospital COVID Prevalence++ /ranks	Median	13039	0.1	5503	0.1	7536	0.1	4.18
	25th		0.1		0.1		0.1	
	75th		0.2		0.2		0.2	
	Mean		0.1		0.1		0.1	
	STD		0.1		0.1		0.1	
	Min		0.0		0.0		0.0	
	Max		0.7		0.7		0.7	
	Missing(%)		0.0		0.0		0.0	
Number of Total Beds++ /ranks	Median	13040	343.0	5503	347.0	7537	342.0	8.14
	25th		240.0		250.0		231.0	
	75th		467.0		471.0		455.0	
	Mean		363.0		371.0		357.2	
	STD		173.5		176.1		171.3	
	Min		34.0		42.0		34.0	
	Max		899.0		899.0		899.0	
	Missing(%)		0.0		0.0		0.0	
Number of ICU Beds++ /ranks	Median	13040	36.0	5503	37.0	7537	34.0	11.69
	25th		20.0		21.0		20.0	
	75th		50.0		52.0		50.0	
	Mean		41.3		43.2		39.9	
	STD		27.0		28.0		26.1	
	Min		0.0		0.0		0.0	
	Max		139.0		139.0		139.0	
	Missing(%)		0.0		0.0		0.0	
Region	West	1414	10.8	547	9.9	867	11.5	11.35

© 2023 Mourad A et al. *JAMA Network Open.*

Variable	Level	Overall (N=13040)		Control (N=5503)		Dexamethasone (N=7537)		Std Diff Score*
	South	11176	85.7	4705	85.5	6471	85.9	
	Midwest	382	2.9	215	3.9	167	2.2	
	Northeast	54	0.4	30	0.5	24	0.3	
	Missing	14	0.1	6	0.1	8	0.1	
Calendar Epoch Intervals++ /ranks	Epoch 10: Q4 2021	244	1.9	119	2.2	125	1.7	11.72
	Epoch 9: Q3 2021	3126	24.0	1272	23.1	1854	24.6	
	Epoch 8: Q2 2021	1040	8.0	366	6.7	674	8.9	
	Epoch 7: Q1 2021	2993	23.0	1195	21.7	1798	23.9	
	Epoch 6: August 24 - December 31, 2020	3580	27.5	1481	26.9	2099	27.8	
	Epoch 5: July 6 - August 23, 2020	1843	14.1	970	17.6	873	11.6	
	Epoch 4: May 20 - July 5, 2020	214	1.6	100	1.8	114	1.5	
Age++ /ranks	Median	13040	66.0	5503	68.0	7537	64.0	14.51
	25th		52.0		53.0		51.0	
	75th		78.0		79.0		76.0	
	Mean		64.0		65.2		63.1	
	STD		17.6		18.1		17.2	
	Min		18.0		18.0		18.0	
	Max		104.0		103.0		104.0	
	Missing(%)		0.0		0.0		0.0	
Age group++ /ranks	3: 70+	5259	40.3	2443	44.4	2816	37.4	12.76
	2: 51-70	4809	36.9	1883	34.2	2926	38.8	
	1: 18-50	2972	22.8	1177	21.4	1795	23.8	
Female	Yes	6379	48.9	2738	49.8	3641	48.3	2.89
	No	6661	51.1	2765	50.2	3896	51.7	

© 2023 Mourad A et al. *JAMA Network Open.*

Variable	Level	Overall (N=13040)		Control (N=5503)		Dexamethasone (N=7537)		Std Diff Score*
Race	Other	2609	20.0	1051	19.1	1558	20.7	5.71
	Black	2901	22.2	1291	23.5	1610	21.4	
	White	7530	57.7	3161	57.4	4369	58.0	
Hispanic	Yes	3121	23.9	1238	22.5	1883	25.0	5.85
	No	9919	76.1	4265	77.5	5654	75.0	
Smoking	Current smoker	935	7.2	483	8.8	452	6.0	16.97
	Former smoker	2680	20.6	1131	20.6	1549	20.6	
	Never smoker	8090	62.0	3214	58.4	4876	64.7	
	Unknown	1335	10.2	675	12.3	660	8.8	
BMI++ /ranks	Median	10968	28.1	4463	27.4	6505	28.6	22.80
	25th		24.5		23.8		25.1	
	75th		32.9		31.9		33.5	
	Mean		29.3		28.4		30.0	
	STD		7.2		6.9		7.3	
	Min		11.6		11.6		12.1	
	Max		76.2		72.1		76.2	
	Missing(%)		15.9		18.9		13.7	
Hypertension	Yes	9634	73.9	4270	77.6	5364	71.2	14.76
	No	3406	26.1	1233	22.4	2173	28.8	
Hyperlipidemia	Yes	6473	49.6	2901	52.7	3572	47.4	10.66
	No	6567	50.4	2602	47.3	3965	52.6	
CAD	Yes	3782	29.0	1808	32.9	1974	26.2	14.65
	No	9258	71.0	3695	67.1	5563	73.8	
MI	Yes	1523	11.7	761	13.8	762	10.1	11.69
	No	11353	87.1	4662	84.7	6691	88.8	

© 2023 Mourad A et al. *JAMA Network Open.*

Variable	Level	Overall (N=13040)		Control (N=5503)		Dexamethasone (N=7537)		Std Diff Score*
	Missing	164	1.3	80	1.5	84	1.1	
Diabetes	Yes	5735	44.0	2615	47.5	3120	41.4	12.35
	No	7305	56.0	2888	52.5	4417	58.6	
Pre-Diabetes	Yes	174	1.3	68	1.2	106	1.4	1.50
	No	12866	98.7	5435	98.8	7431	98.6	
Obesity	Yes	3891	29.8	1505	27.3	2386	31.7	9.46
	No	9149	70.2	3998	72.7	5151	68.3	
Asthma	Yes	1165	8.9	401	7.3	764	10.1	10.12
	No	11875	91.1	5102	92.7	6773	89.9	
Autoimmune	Yes	1363	10.5	655	11.9	708	9.4	8.14
	No	11677	89.5	4848	88.1	6829	90.6	
COPD	Yes	3135	24.0	1259	22.9	1876	24.9	4.72
	No	9905	76.0	4244	77.1	5661	75.1	
Sarcoidosis	Yes	27	0.2	9	0.2	18	0.2	1.68
	No	13013	99.8	5494	99.8	7519	99.8	
ILD	Yes	38	0.3	16	0.3	22	0.3	0.02
	No	13002	99.7	5487	99.7	7515	99.7	
Stroke	Yes	485	3.7	287	5.2	198	2.6	13.52
	No	12391	95.0	5136	93.3	7255	96.3	
	Missing	164	1.3	80	1.5	84	1.1	
PE	Yes	371	2.8	168	3.1	203	2.7	2.23
	No	12505	95.9	5255	95.5	7250	96.2	

© 2023 Mourad A et al. *JAMA Network Open.*

Variable	Level	Overall (N=13040)		Control (N=5503)		Dexamethasone (N=7537)		Std Diff Score*
	Missing	164	1.3	80	1.5	84	1.1	
HIV/AIDS	Yes	63	0.5	37	0.7	26	0.3	4.60
	No	12977	99.5	5466	99.3	7511	99.7	
Lymphoma/Leukemia	Yes	199	1.5	83	1.5	116	1.5	0.25
	No	12841	98.5	5420	98.5	7421	98.5	
Solid tumors	Yes	865	6.6	457	8.3	408	5.4	11.46
	No	12175	93.4	5046	91.7	7129	94.6	
CKD	Yes	6883	52.8	3269	59.4	3614	48.0	23.12
	No	6157	47.2	2234	40.6	3923	52.0	
Prior Dialysis	Yes	46	0.4	31	0.6	15	0.2	5.91
	No	12994	99.6	5472	99.4	7522	99.8	
Prior Revascularization	Yes	1556	11.9	741	13.5	815	10.8	8.23
	No	11414	87.5	4726	85.9	6688	88.7	
	Missing	70	0.5	36	0.7	34	0.5	
Charlson Comorbidity Index	2: 2+ comorbidities	6270	48.1	3073	55.8	3197	42.4	29.37
	1: 1 comorbidity	3360	25.8	1296	23.6	2064	27.4	
	0: 0 conditions	3246	24.9	1054	19.2	2192	29.1	
	Missing	164	1.3	80	1.5	84	1.1	
Baseline Spo2++ /ranks	Median	13026	94.0	5497	95.0	7529	94.0	52.36
	25th		92.0		93.0		92.0	
	75th		96.0		96.0		95.0	
	Mean		93.8		94.5		93.3	
	STD		3.1		3.1		3.1	
	Min		60.0		60.0		61.0	

© 2023 Mourad A et al. *JAMA Network Open.*

Variable	Level	Overall (N=13040)		Control (N=5503)		Dexamethasone (N=7537)		Std Diff Score*
	Max		100.0		100.0		100.0	
	Missing(%)		0.1		0.1		0.1	
Spo2++ /ranks	Median	13029	93.0	5497	94.0	7532	93.0	15.53
	25th		92.0		92.0		92.0	
	75th		95.0		95.0		95.0	
	Mean		93.1		93.3		93.0	
	STD		3.1		3.2		3.0	
	Min		61.0		62.0		61.0	
	Max		100.0		100.0		100.0	
	Missing(%)		0.1		0.1		0.1	
FiO2++ /ranks	Median	13040	21.0	5503	21.0	7537	21.0	.
	25th		21.0		21.0		21.0	
	75th		21.0		21.0		21.0	
	Mean		21.0		21.0		21.0	
	STD		0.0		0.0		0.0	
	Min		21.0		21.0		21.0	
	Max		21.0		21.0		21.0	
	Missing(%)		0.0		0.0		0.0	
S/F ratio++ /ranks	Median	13029	4.4	5497	4.5	7532	4.4	15.53
	25th		4.4		4.4		4.4	
	75th		4.5		4.5		4.5	
	Mean		4.4		4.4		4.4	
	STD		0.1		0.2		0.1	
	Min		2.9		3.0		2.9	
	Max		4.8		4.8		4.8	
	Missing(%)		0.1		0.1		0.1	
eGFR++ /ranks	Median	10848	78.4	4402	71.8	6446	82.6	18.50
	25th		47.3		41.3		51.3	

© 2023 Mourad A et al. *JAMA Network Open.*

Variable	Level	Overall (N=13040)		Control (N=5503)		Dexamethasone (N=7537)		Std Diff Score*
	75th		117.5		112.5		120.0	
	Mean		76.9		72.7		79.7	
	STD		35.7		36.8		34.7	
	Min		2.1		2.1		2.4	
	Max		120.0		120.0		120.0	
	Missing(%)		16.8		20.0		14.5	
Time since admission++ /ranks	Median	13040	1.0	5503	1.0	7537	1.0	.
	25th		1.0		1.0		1.0	
	75th		1.0		1.0		1.0	
	Mean		1.0		1.0		1.0	
	STD		0.0		0.0		0.0	
	Min		1.0		1.0		1.0	
	Max		1.0		1.0		1.0	
	Missing(%)		0.0		0.0		0.0	
ICU	Yes	721	5.5	430	7.8	291	3.9	16.92
	No	12319	94.5	5073	92.2	7246	96.1	
Anticoagulants (prophylactic)	Yes	5750	44.1	2720	49.4	3030	40.2	18.63
	No	7290	55.9	2783	50.6	4507	59.8	
Anticoagulants (treatment)	Yes	1909	14.6	891	16.2	1018	13.5	7.55
	No	11131	85.4	4612	83.8	6519	86.5	
Remdesivir	Yes	1047	8.0	124	2.3	923	12.2	39.27
	No	11993	92.0	5379	97.7	6614	87.8	
Vasopressors	Yes	20	0.2	15	0.3	5	0.1	5.02
	No	13020	99.8	5488	99.7	7532	99.9	
Tocilizumab	Yes	1	0.0	0	0.0	1	0.0	1.63

© 2023 Mourad A et al. *JAMA Network Open.*

Variable	Level	Overall (N=13040)		Control (N=5503)		Dexamethasone (N=7537)		Std Diff Score*
	No	13039	100.0	5503	100.0	7536	100.0	
Baricitinib	Yes	7	0.1	0	0.0	7	0.1	4.31
	No	13033	99.9	5503	100.0	7530	99.9	
Bamlanivimab/ etesevimab	Yes	8	0.1	3	0.1	5	0.1	0.48
	No	13032	99.9	5500	99.9	7532	99.9	
Casirivimab/ Imdevimab	Yes	39	0.3	23	0.4	16	0.2	3.67
	No	13001	99.7	5480	99.6	7521	99.8	
Azithromycin	Yes	6959	53.4	2157	39.2	4802	63.7	50.60
	No	6081	46.6	3346	60.8	2735	36.3	
Antibiotics	Yes	10055	77.1	3940	71.6	6115	81.1	22.59
	No	2985	22.9	1563	28.4	1422	18.9	

* Standardized difference scores measure the effect size between Control and Dexamethasone (All Standardized difference scores have been multiplied by 100).
++ /ranks indicates standardized difference score was calculated using rank statistics for the row variable.

© 2023 Mourad A et al. *JAMA Network Open.*

eTable 2. Characteristics of Patients Eligible for the Supplemental Oxygen Cohort

Variable	Level	Overall (N=56368)		Control (N=7789)		Dexamethasone (N=48579)		Std Diff Score*
**Demographics**								
Hospital COVID Prevalence++ /ranks	Median	56364	0.1	7789	0.1	48575	0.1	6.34
	25th		0.1		0.1		0.1	
	75th		0.2		0.2		0.2	
	Mean		0.1		0.1		0.1	
	STD		0.1		0.1		0.1	
	Min		0.0		0.0		0.0	
	Max		0.9		0.7		0.9	
	Missing(%)		0.0		0.0		0.0	
Number of Total Beds++ /ranks	Median	56368	319.0	7789	323.0	48579	319.0	4.08
	25th		227.0		240.0		227.0	
	75th		449.0		455.0		449.0	
	Mean		347.7		351.6		347.1	
	STD		177.6		171.1		178.6	
	Min		34.0		42.0		34.0	
	Max		899.0		899.0		899.0	
	Missing(%)		0.0		0.0		0.0	
Number of ICU Beds++ /ranks	Median	56368	34.0	7789	36.0	48579	34.0	2.18
	25th		20.0		20.0		20.0	
	75th		48.0		48.0		48.0	
	Mean		40.0		40.1		40.0	
	STD		27.2		26.4		27.4	
	Min		0.0		0.0		0.0	
	Max		139.0		139.0		139.0	
	Missing(%)		0.0		0.0		0.0	
Region	West	9206	16.3	864	11.1	8342	17.2	19.50

© 2023 Mourad A et al. *JAMA Network Open.*

Variable	Level	Overall (N=56368)		Control (N=7789)		Dexamethasone (N=48579)		Std Diff Score*
	South	45088	80.0	6522	83.7	38566	79.4	
	Midwest	1830	3.2	379	4.9	1451	3.0	
	Northeast	169	0.3	19	0.2	150	0.3	
	Missing	75	0.1	5	0.1	70	0.1	
Calendar Epoch Intervals++ /ranks	Epoch 10: Q4 2021	1465	2.6	178	2.3	1287	2.6	19.82
	Epoch 9: Q3 2021	15819	28.1	1890	24.3	13929	28.7	
	Epoch 8: Q2 2021	4389	7.8	485	6.2	3904	8.0	
	Epoch 7: Q1 2021	12128	21.5	1608	20.6	10520	21.7	
	Epoch 6: August 24 - December 31, 2020	15226	27.0	1961	25.2	13265	27.3	
	Epoch 5: July 6 - August 23, 2020	6602	11.7	1501	19.3	5101	10.5	
	Epoch 4: May 20 - July 5, 2020	739	1.3	166	2.1	573	1.2	
Age++ /ranks	Median	56368	65.0	7789	70.0	48579	64.0	35.09
	25th		52.0		58.0		51.0	
	75th		76.0		81.0		75.0	
	Mean		63.4		68.2		62.7	
	STD		16.6		16.5		16.5	
	Min		18.0		18.0		18.0	
	Max		109.0		103.0		109.0	
	Missing(%)		0.0		0.0		0.0	
Age group++ /ranks	3: 70+	20807	36.9	3874	49.7	16933	34.9	31.60
	2: 51-70	22807	40.5	2715	34.9	20092	41.4	
	1: 18-50	12754	22.6	1200	15.4	11554	23.8	
Female	Yes	26619	47.2	4019	51.6	22600	46.5	10.17
	No	29749	52.8	3770	48.4	25979	53.5	

© 2023 Mourad A et al. *JAMA Network Open.*

Variable	Level	Overall (N=56368)		Control (N=7789)		Dexamethasone (N=48579)		Std Diff Score*
Race	Other	12802	22.7	1358	17.4	11444	23.6	18.38
	Black	8610	15.3	1530	19.6	7080	14.6	
	White	34956	62.0	4901	62.9	30055	61.9	
Hispanic	Yes	14401	25.5	1691	21.7	12710	26.2	10.45
	No	41967	74.5	6098	78.3	35869	73.8	
Smoking	Current smoker	2823	5.0	523	6.7	2300	4.7	20.56
	Former smoker	13042	23.1	1863	23.9	11179	23.0	
	Never smoker	34712	61.6	4246	54.5	30466	62.7	
	Unknown	5791	10.3	1157	14.9	4634	9.5	
BMI++ /ranks	Median	48895	30.2	6465	28.5	42430	30.4	25.35
	25th		26.0		24.5		26.4	
	75th		35.6		34.0		35.8	
	Mean		31.6		30.1		31.8	
	STD		8.1		8.1		8.1	
	Min		8.0		9.2		8.0	
	Max		137.3		92.6		137.3	
	Missing(%)		13.3		17.0		12.7	
Hypertension	Yes	40177	71.3	6332	81.3	33845	69.7	27.27
	No	16191	28.7	1457	18.7	14734	30.3	
Hyperlipidemia	Yes	26733	47.4	4426	56.8	22307	45.9	21.95
	No	29635	52.6	3363	43.2	26272	54.1	
CAD	Yes	15224	27.0	2979	38.2	12245	25.2	28.30
	No	41144	73.0	4810	61.8	36334	74.8	
MI	Yes	6315	11.2	1322	17.0	4993	10.3	19.74
	No	49103	87.1	6346	81.5	42757	88.0	

© 2023 Mourad A et al. *JAMA Network Open.*

Variable	Level	Overall (N=56368)		Control (N=7789)		Dexamethasone (N=48579)		Std Diff Score*
	Missing	950	1.7	121	1.6	829	1.7	
Diabetes	Yes	25079	44.5	3926	50.4	21153	43.5	13.78
	No	31289	55.5	3863	49.6	27426	56.5	
Pre-Diabetes	Yes	1071	1.9	83	1.1	988	2.0	7.84
	No	55297	98.1	7706	98.9	47591	98.0	
Obesity	Yes	23033	40.9	2937	37.7	20096	41.4	7.49
	No	33335	59.1	4852	62.3	28483	58.6	
Asthma	Yes	5172	9.2	706	9.1	4466	9.2	0.45
	No	51196	90.8	7083	90.9	44113	90.8	
Autoimmune	Yes	4604	8.2	931	12.0	3673	7.6	14.84
	No	51764	91.8	6858	88.0	44906	92.4	
COPD	Yes	15619	27.7	2704	34.7	12915	26.6	17.70
	No	40749	72.3	5085	65.3	35664	73.4	
Sarcoidosis	Yes	153	0.3	15	0.2	138	0.3	1.88
	No	56215	99.7	7774	99.8	48441	99.7	
ILD	Yes	241	0.4	44	0.6	197	0.4	2.29
	No	56127	99.6	7745	99.4	48382	99.6	
Stroke	Yes	1516	2.7	386	5.0	1130	2.3	14.17
	No	53902	95.6	7282	93.5	46620	96.0	
	Missing	950	1.7	121	1.6	829	1.7	
PE	Yes	1924	3.4	345	4.4	1579	3.3	6.16
	No	53494	94.9	7323	94.0	46171	95.0	

© 2023 Mourad A et al. *JAMA Network Open.*

Variable	Level	Overall (N=56368)		Control (N=7789)		Dexamethasone (N=48579)		Std Diff Score*
	Missing	950	1.7	121	1.6	829	1.7	
HIV/AIDS	Yes	156	0.3	35	0.4	121	0.2	3.40
	No	56212	99.7	7754	99.6	48458	99.8	
Lymphoma/Leukemia	Yes	753	1.3	127	1.6	626	1.3	2.85
	No	55615	98.7	7662	98.4	47953	98.7	
Solid tumors	Yes	2965	5.3	635	8.2	2330	4.8	13.67
	No	53403	94.7	7154	91.8	46249	95.2	
CKD	Yes	29369	52.1	4930	63.3	24439	50.3	26.45
	No	26999	47.9	2859	36.7	24140	49.7	
Prior Dialysis	Yes	156	0.3	51	0.7	105	0.2	6.67
	No	56212	99.7	7738	99.3	48474	99.8	
Prior Revascularization	Yes	6208	11.0	1233	15.8	4975	10.2	16.75
	No	49631	88.0	6482	83.2	43149	88.8	
	Missing	529	0.9	74	1.0	455	0.9	
Charlson Comorbidity Index	2: 2+ comorbidities	24731	43.9	4781	61.4	19950	41.1	43.96
	1: 1 comorbidity	15203	27.0	1671	21.5	13532	27.9	
	0: 0 conditions	15484	27.5	1216	15.6	14268	29.4	
	Missing	950	1.7	121	1.6	829	1.7	
Baseline Spo2++ /ranks	Median	55939	91.0	7761	93.0	48178	90.0	59.79
	25th		88.0		90.0		87.0	
	75th		93.0		95.0		93.0	
	Mean		89.6		91.9		89.2	
	STD		5.6		4.7		5.6	
	Min		60.0		60.0		60.0	

© 2023 Mourad A et al. *JAMA Network Open.*

Variable	Level	Overall (N=56368)		Control (N=7789)		Dexamethasone (N=48579)		Std Diff Score*
	Max		100.0		100.0		100.0	
	Missing(%)		0.8		0.4		0.8	
Spo2++ /ranks	Median	55934	90.0	7753	92.0	48181	90.0	41.84
	25th		88.0		90.0		87.0	
	75th		93.0		94.0		92.0	
	Mean		89.4		91.0		89.1	
	STD		5.4		4.5		5.4	
	Min		60.0		60.0		60.0	
	Max		100.0		100.0		100.0	
	Missing(%)		0.8		0.5		0.8	
FiO2++ /ranks	Median	56368	36.0	7789	32.0	48579	36.0	33.12
	25th		28.0		28.0		28.0	
	75th		48.0		38.0		50.0	
	Mean		45.3		39.5		46.3	
	STD		24.6		20.5		25.0	
	Min		21.0		21.0		21.0	
	Max		100.0		100.0		100.0	
	Missing(%)		0.0		0.0		0.0	
S/F ratio++ /ranks	Median	55934	2.6	7753	2.9	48181	2.6	37.27
	25th		1.9		2.3		1.8	
	75th		3.2		3.3		3.1	
	Mean		2.4		2.7		2.4	
	STD		0.9		0.8		0.9	
	Min		0.6		0.6		0.6	
	Max		4.8		4.7		4.8	
	Missing(%)		0.8		0.5		0.8	
eGFR++ /ranks	Median	48422	86.8	6409	69.9	42013	89.0	31.83
	25th		53.7		39.8		56.3	

© 2023 Mourad A et al. *JAMA Network Open.*

Variable	Level	Overall (N=56368)		Control (N=7789)		Dexamethasone (N=48579)		Std Diff Score*
	75th		120.0		112.0		120.0	
	Mean		82.1		71.6		83.7	
	STD		34.9		37.3		34.2	
	Min		2.2		3.0		2.2	
	Max		120.0		120.0		120.0	
	Missing(%)		14.1		17.7		13.5	
Oxygen L/min++ /ranks	1: greater than 15 L/min	5460	9.7	382	4.9	5078	10.5	20.95
	0: less than or equal to 15 L/min	50908	90.3	7407	95.1	43501	89.5	
Time since admission++ /ranks	Median	56368	1.0	7789	1.0	48579	1.0	52.91
	25th		1.0		1.0		1.0	
	75th		1.0		1.0		1.0	
	Mean		1.1		1.4		1.0	
	STD		0.6		1.1		0.4	
	Min		1.0		1.0		1.0	
	Max		36.0		19.0		36.0	
	Missing(%)		0.0		0.0		0.0	
ICU	Yes	6398	11.4	1211	15.5	5187	10.7	14.47
	No	49970	88.6	6578	84.5	43392	89.3	
Anticoagulants (prophylactic)	Yes	25745	45.7	3802	48.8	21943	45.2	7.30
	No	30623	54.3	3987	51.2	26636	54.8	
Anticoagulants (treatment)	Yes	11145	19.8	1712	22.0	9433	19.4	6.33
	No	45223	80.2	6077	78.0	39146	80.6	
Remdesivir	Yes	20284	36.0	1454	18.7	18830	38.8	45.55
	No	36084	64.0	6335	81.3	29749	61.2	

© 2023 Mourad A et al. *JAMA Network Open.*

Variable	Level	Overall (N=56368)		Control (N=7789)		Dexamethasone (N=48579)		Std Diff Score*
Vasopressors	Yes	191	0.3	66	0.8	125	0.3	7.97
	No	56177	99.7	7723	99.2	48454	99.7	
Tocilizumab	Yes	262	0.5	15	0.2	247	0.5	5.35
	No	56106	99.5	7774	99.8	48332	99.5	
Baricitinib	Yes	311	0.6	23	0.3	288	0.6	4.48
	No	56057	99.4	7766	99.7	48291	99.4	
Bamlanivimab/Etesevimab	Yes	8	0.0	1	0.0	7	0.0	0.13
	No	56360	100.0	7788	100.0	48572	100.0	
Casirivimab/Imdevimab	Yes	82	0.1	19	0.2	63	0.1	2.65
	No	56286	99.9	7770	99.8	48516	99.9	
Azithromycin	Yes	33421	59.3	3583	46.0	29838	61.4	31.30
	No	22947	40.7	4206	54.0	18741	38.6	
Antibiotics	Yes	45014	79.9	6159	79.1	38855	80.0	2.26
	No	11354	20.1	1630	20.9	9724	20.0	

* Standardized difference scores measure the effect size between Control and Dexamethasone (All Standardized difference scores have been multiplied by 100).
++ /ranks indicates standardized difference score was calculated using rank statistics for the row variable.

© 2023 Mourad A et al. *JAMA Network Open.*

eTable 3. Characteristics of Patients Eligible for the Noninvasive Positive Pressure Ventilation (NIPPV) Cohort

Variable	Level	Overall (N=7618)		Control (N=792)		Dexamethasone (N=6826)		Std Diff Score*
**Demographics**								
Hospital COVID Prevalence++ /ranks	Median	7618	0.1	792	0.1	6826	0.1	3.33
	25th		0.1		0.1		0.1	
	75th		0.2		0.2		0.2	
	Mean		0.2		0.2		0.2	
	STD		0.1		0.1		0.1	
	Min		0.0		0.0		0.0	
	Max		0.7		0.6		0.7	
	Missing(%)		0.0		0.0		0.0	
Number of Total Beds++ /ranks	Median	7618	319.0	792	298.5	6826	319.0	7.33
	25th		227.0		221.0		227.0	
	75th		447.0		450.0		447.0	
	Mean		337.4		328.6		338.4	
	STD		157.8		160.5		157.4	
	Min		50.0		61.0		50.0	
	Max		899.0		899.0		899.0	
	Missing(%)		0.0		0.0		0.0	
Number of ICU Beds++ /ranks	Median	7618	34.0	792	32.0	6826	36.0	4.46
	25th		20.0		19.0		20.0	
	75th		48.0		48.0		48.0	
	Mean		38.0		36.8		38.2	
	STD		23.7		22.5		23.8	
	Min		0.0		6.0		0.0	
	Max		139.0		139.0		139.0	
	Missing(%)		0.0		0.0		0.0	
Region	West	826	10.8	39	4.9	787	11.5	36.96

© 2023 Mourad A et al. *JAMA Network Open.*

Variable	Level	Overall (N=7618)		Control (N=792)		Dexamethasone (N=6826)		Std Diff Score*
	South	6389	83.9	655	82.7	5734	84.0	
	Midwest	390	5.1	98	12.4	292	4.3	
	Missing	13	0.2	0	0.0	13	0.2	
Calendar Epoch Intervals++ /ranks	Epoch 10: Q4 2021	144	1.9	14	1.8	130	1.9	2.40
	Epoch 9: Q3 2021	2460	32.3	267	33.7	2193	32.1	
	Epoch 8: Q2 2021	518	6.8	51	6.4	467	6.8	
	Epoch 7: Q1 2021	1534	20.1	132	16.7	1402	20.5	
	Epoch 6: August 24 - December 31, 2020	2005	26.3	211	26.6	1794	26.3	
	Epoch 5: July 6 - August 23, 2020	873	11.5	113	14.3	760	11.1	
	Epoch 4: May 20 - July 5, 2020	84	1.1	4	0.5	80	1.2	
Age++ /ranks	Median	7618	65.0	792	68.0	6826	65.0	22.65
	25th		54.0		58.0		54.0	
	75th		75.0		77.0		75.0	
	Mean		64.1		67.2		63.8	
	STD		14.9		14.1		14.9	
	Min		18.0		23.0		18.0	
	Max		102.0		99.0		102.0	
	Missing(%)		0.0		0.0		0.0	
Age group++ /ranks	3: 70+	2732	35.9	342	43.2	2390	35.0	21.64
	2: 51-70	3466	45.5	354	44.7	3112	45.6	
	1: 18-50	1420	18.6	96	12.1	1324	19.4	
Female	Yes	3113	40.9	369	46.6	2744	40.2	12.92
	No	4505	59.1	423	53.4	4082	59.8	
Race	Other	1803	23.7	121	15.3	1682	24.6	23.62

© 2023 Mourad A et al. *JAMA Network Open.*

Variable	Level	Overall (N=7618)		Control (N=792)		Dexamethasone (N=6826)		Std Diff Score*
	Black	1143	15.0	135	17.0	1008	14.8	
	White	4672	61.3	536	67.7	4136	60.6	
Hispanic	Yes	1866	24.5	149	18.8	1717	25.2	15.36
	No	5752	75.5	643	81.2	5109	74.8	
Smoking	Current smoker	401	5.3	42	5.3	359	5.3	9.76
	Former smoker	1823	23.9	214	27.0	1609	23.6	
	Never smoker	4114	54.0	395	49.9	3719	54.5	
	Unknown	1280	16.8	141	17.8	1139	16.7	
BMI++ /ranks	Median	6539	32.5	649	32.0	5890	32.6	3.72
	25th		27.5		26.9		27.5	
	75th		38.9		39.3		38.8	
	Mean		34.0		33.8		34.0	
	STD		9.5		10.2		9.5	
	Min		11.0		13.7		11.0	
	Max		157.7		152.6		157.7	
	Missing(%)		14.2		18.1		13.7	
Hypertension	Yes	6096	80.0	679	85.7	5417	79.4	16.85
	No	1522	20.0	113	14.3	1409	20.6	
Hyperlipidemia	Yes	3956	51.9	490	61.9	3466	50.8	22.51
	No	3662	48.1	302	38.1	3360	49.2	
CAD	Yes	3013	39.6	358	45.2	2655	38.9	12.80
	No	4605	60.4	434	54.8	4171	61.1	
MI	Yes	1512	19.8	185	23.4	1327	19.4	9.91
	No	5992	78.7	592	74.7	5400	79.1	
	Missing	114	1.5	15	1.9	99	1.5	

© 2023 Mourad A et al. *JAMA Network Open.*

Variable	Level	Overall (N=7618)		Control (N=792)		Dexamethasone (N=6826)		Std Diff Score*
Diabetes	Yes	4335	56.9	480	60.6	3855	56.5	8.39
	No	3283	43.1	312	39.4	2971	43.5	
Pre-Diabetes	Yes	129	1.7	7	0.9	122	1.8	7.88
	No	7489	98.3	785	99.1	6704	98.2	
Obesity	Yes	4458	58.5	471	59.5	3987	58.4	2.16
	No	3160	41.5	321	40.5	2839	41.6	
Asthma	Yes	729	9.6	81	10.2	648	9.5	2.46
	No	6889	90.4	711	89.8	6178	90.5	
Autoimmune	Yes	631	8.3	85	10.7	546	8.0	9.39
	No	6987	91.7	707	89.3	6280	92.0	
COPD	Yes	2700	35.4	339	42.8	2361	34.6	16.93
	No	4918	64.6	453	57.2	4465	65.4	
Sarcoidosis	Yes	14	0.2	1	0.1	13	0.2	1.61
	No	7604	99.8	791	99.9	6813	99.8	
ILD	Yes	37	0.5	6	0.8	31	0.5	3.91
	No	7581	99.5	786	99.2	6795	99.5	
Stroke	Yes	260	3.4	33	4.2	227	3.3	4.56
	No	7244	95.1	744	93.9	6500	95.2	
	Missing	114	1.5	15	1.9	99	1.5	
PE	Yes	388	5.1	49	6.2	339	5.0	5.48
	No	7116	93.4	728	91.9	6388	93.6	
	Missing	114	1.5	15	1.9	99	1.5	

© 2023 Mourad A et al. *JAMA Network Open.*

Variable	Level	Overall (N=7618)		Control (N=792)		Dexamethasone (N=6826)		Std Diff Score*
HIV/AIDS	Yes	19	0.2	2	0.3	17	0.2	0.07
	No	7599	99.8	790	99.7	6809	99.8	
Lymphoma/Leukemia	Yes	103	1.4	13	1.6	90	1.3	2.67
	No	7515	98.6	779	98.4	6736	98.7	
Solid tumors	Yes	393	5.2	51	6.4	342	5.0	6.15
	No	7225	94.8	741	93.6	6484	95.0	
CKD	Yes	5627	73.9	597	75.4	5030	73.7	3.88
	No	1991	26.1	195	24.6	1796	26.3	
Prior Dialysis	Yes	18	0.2	3	0.4	15	0.2	2.91
	No	7600	99.8	789	99.6	6811	99.8	
Prior Revascularization	Yes	1040	13.7	144	18.2	896	13.1	14.25
	No	6493	85.2	636	80.3	5857	85.8	
	Missing	85	1.1	12	1.5	73	1.1	
Charlson Comorbidity Index	2: 2+ comorbidities	4535	59.5	532	67.2	4003	58.6	18.75
	1: 1 comorbidity	1777	23.3	147	18.6	1630	23.9	
	0: 0 conditions	1192	15.6	98	12.4	1094	16.0	
	Missing	114	1.5	15	1.9	99	1.5	
Baseline Spo2++ /ranks	Median	7097	87.0	765	90.0	6332	87.0	46.18
	25th		80.0		85.0		80.0	
	75th		92.0		93.0		91.0	
	Mean		85.0		88.1		84.6	
	STD		8.8		7.8		8.8	
	Min		60.0		60.0		60.0	
	Max		100.0		100.0		100.0	

© 2023 Mourad A et al. *JAMA Network Open.*

Variable	Level	Overall (N=7618)		Control (N=792)		Dexamethasone (N=6826)		Std Diff Score*
	Missing(%)		6.8		3.4		7.2	
Spo2++ /ranks	Median	7121	87.0	772	89.0	6349	86.0	44.18
	25th		80.0		86.0		80.0	
	75th		91.0		92.0		91.0	
	Mean		84.9		87.9		84.5	
	STD		8.3		6.3		8.5	
	Min		60.0		60.0		60.0	
	Max		100.0		100.0		100.0	
	Missing(%)		6.5		2.5		7.0	
FiO2++ /ranks	Median	7490	100.0	781	98.0	6709	100.0	28.92
	25th		70.0		50.0		70.0	
	75th		100.0		100.0		100.0	
	Mean		84.1		76.8		84.9	
	STD		23.2		27.9		22.5	
	Min		21.0		21.0		21.0	
	Max		100.0		100.0		100.0	
	Missing(%)		1.7		1.4		1.7	
S/F ratio++ /ranks	Median	6996	0.9	761	0.9	6235	0.9	38.80
	25th		0.8		0.9		0.8	
	75th		1.3		1.8		1.3	
	Mean		1.2		1.4		1.1	
	STD		0.6		0.9		0.6	
	Min		0.6		0.6		0.6	
	Max		4.6		4.6		4.6	
	Missing(%)		8.2		3.9		8.7	
eGFR++ /ranks	Median	6480	79.7	647	75.2	5833	80.1	6.71
	25th		47.2		43.2		47.7	
	75th		120.0		120.0		120.0	

© 2023 Mourad A et al. *JAMA Network Open.*

Variable	Level	Overall (N=7618)		Control (N=792)		Dexamethasone (N=6826)		Std Diff Score*
	Mean		77.5		75.0		77.8	
	STD		36.1		37.1		35.9	
	Min		2.1		3.7		2.1	
	Max		120.0		120.0		120.0	
	Missing(%)		14.9		18.3		14.5	
Time since admission++ /ranks	Median	7618	1.0	792	1.0	6826	1.0	101.96
	25th		1.0		1.0		1.0	
	75th		1.0		3.0		1.0	
	Mean		1.2		2.3		1.1	
	STD		1.1		2.5		0.6	
	Min		1.0		1.0		1.0	
	Max		26.0		26.0		15.0	
	Missing(%)		0.0		0.0		0.0	
ICU	Yes	3354	44.0	337	42.6	3017	44.2	3.33
	No	4264	56.0	455	57.4	3809	55.8	
Anticoagulants (prophylactic)	Yes	3043	39.9	402	50.8	2641	38.7	24.45
	No	4575	60.1	390	49.2	4185	61.3	
Anticoagulants (treatment)	Yes	1983	26.0	324	40.9	1659	24.3	35.99
	No	5635	74.0	468	59.1	5167	75.7	
Remdesivir	Yes	2937	38.6	317	40.0	2620	38.4	3.37
	No	4681	61.4	475	60.0	4206	61.6	
Vasopressors	Yes	75	1.0	15	1.9	60	0.9	8.69
	No	7543	99.0	777	98.1	6766	99.1	
Tocilizumab	Yes	210	2.8	30	3.8	180	2.6	6.53
	No	7408	97.2	762	96.2	6646	97.4	

© 2023 Mourad A et al. *JAMA Network Open.*

Variable	Level	Overall (N=7618)		Control (N=792)		Dexamethasone (N=6826)		Std Diff Score*
Baricitinib	Yes	147	1.9	22	2.8	125	1.8	6.31
	No	7471	98.1	770	97.2	6701	98.2	
Bamlanivimab/Etesevimab	No	7618	100.0	792	100.0	6826	100.0	.
Casirivimab/Imdevimab	Yes	4	0.1	1	0.1	3	0.0	2.82
	No	7614	99.9	791	99.9	6823	100.0	
Azithromycin	Yes	4969	65.2	450	56.8	4519	66.2	19.38
	No	2649	34.8	342	43.2	2307	33.8	
Antibiotics	Yes	6688	87.8	666	84.1	6022	88.2	11.98
	No	930	12.2	126	15.9	804	11.8	

* Standardized difference scores measure the effect size between Control and Dexamethasone (All Standardized difference scores have been multiplied by 100).
++ /ranks indicates standardized difference score was calculated using rank statistics for the row variable.

© 2023 Mourad A et al. *JAMA Network Open.*

eTable 4. Characteristics of Patients Eligible for the Mechanical Ventilation (MV) or Extracorporeal Membrane Oxygenation (ECMO) Cohort

Variable	Level	Overall (N=3673)		Control (N=1013)		Dexamethasone (N=2660)		Std Diff Score*
**Demographics**								
Hospital COVID Prevalence++ /ranks	Median	3673	0.1	1013	0.1	2660	0.1	7.43
	25th		0.1		0.1		0.1	
	75th		0.2		0.2		0.2	
	Mean		0.1		0.1		0.1	
	STD		0.1		0.1		0.1	
	Min		0.0		0.0		0.0	
	Max		0.7		0.6		0.7	
	Missing(%)		0.0		0.0		0.0	
Number of Total Beds++ /ranks	Median	3673	346.0	1013	347.0	2660	346.0	1.74
	25th		255.0		250.0		256.0	
	75th		471.0		482.0		470.0	
	Mean		378.3		375.0		379.5	
	STD		182.8		181.7		183.2	
	Min		51.0		61.0		51.0	
	Max		899.0		899.0		899.0	
	Missing(%)		0.0		0.0		0.0	
Number of ICU Beds++ /ranks	Median	3673	39.0	1013	40.0	2660	38.0	1.13
	25th		24.0		24.0		24.0	
	75th		56.0		54.0		56.0	
	Mean		45.3		45.1		45.4	
	STD		28.6		28.0		28.8	
	Min		4.0		4.0		4.0	
	Max		139.0		139.0		139.0	
	Missing(%)		0.0		0.0		0.0	
Region	West	655	17.8	102	10.1	553	20.8	35.75

© 2023 Mourad A et al. *JAMA Network Open.*

Variable	Level	Overall (N=3673)		Control (N=1013)		Dexamethasone (N=2660)		Std Diff Score*
	South	2875	78.3	837	82.6	2038	76.6	
	Midwest	143	3.9	74	7.3	69	2.6	
Calendar Epoch Intervals++ /ranks	Epoch 10: Q4 2021	86	2.3	35	3.5	51	1.9	7.38
	Epoch 9: Q3 2021	929	25.3	269	26.6	660	24.8	
	Epoch 8: Q2 2021	248	6.8	82	8.1	166	6.2	
	Epoch 7: Q1 2021	747	20.3	198	19.5	549	20.6	
	Epoch 6: August 24 - December 31, 2020	1052	28.6	249	24.6	803	30.2	
	Epoch 5: July 6 - August 23, 2020	560	15.2	167	16.5	393	14.8	
	Epoch 4: May 20 - July 5, 2020	51	1.4	13	1.3	38	1.4	
Age++ /ranks	Median	3673	63.0	1013	64.0	2660	63.0	2.52
	25th		52.0		50.0		53.0	
	75th		74.0		74.0		73.0	
	Mean		61.8		61.2		62.0	
	STD		16.0		17.2		15.5	
	Min		18.0		18.0		19.0	
	Max		98.0		97.0		98.0	
	Missing(%)		0.0		0.0		0.0	
Age group++ /ranks	3: 70+	1175	32.0	336	33.2	839	31.5	2.73
	2: 51-70	1644	44.8	412	40.7	1232	46.3	
	1: 18-50	854	23.3	265	26.2	589	22.1	
Female	Yes	1495	40.7	420	41.5	1075	40.4	2.13
	No	2178	59.3	593	58.5	1585	59.6	
Race	Other	1033	28.1	222	21.9	811	30.5	19.61
	Black	576	15.7	175	17.3	401	15.1	

© 2023 Mourad A et al. *JAMA Network Open.*

Variable	Level	Overall (N=3673)		Control (N=1013)		Dexamethasone (N=2660)		Std Diff Score*
	White	2064	56.2	616	60.8	1448	54.4	
Hispanic	Yes	952	25.9	247	24.4	705	26.5	4.87
	No	2721	74.1	766	75.6	1955	73.5	
Smoking	Current smoker	267	7.3	95	9.4	172	6.5	16.43
	Former smoker	521	14.2	168	16.6	353	13.3	
	Never smoker	1303	35.5	358	35.3	945	35.5	
	Unknown	1582	43.1	392	38.7	1190	44.7	
BMI++ /ranks	Median	3105	30.0	844	29.6	2261	30.2	9.62
	25th		25.7		25.3		25.8	
	75th		36.0		34.9		36.5	
	Mean		31.6		30.9		31.8	
	STD		8.7		8.3		8.8	
	Min		9.7		14.4		9.7	
	Max		68.1		68.1		66.0	
	Missing(%)		15.5		16.7		15.0	
Hypertension	Yes	2838	77.3	789	77.9	2049	77.0	2.05
	No	835	22.7	224	22.1	611	23.0	
Hyperlipidemia	Yes	1755	47.8	493	48.7	1262	47.4	2.45
	No	1918	52.2	520	51.3	1398	52.6	
CAD	Yes	1607	43.8	429	42.3	1178	44.3	3.91
	No	2066	56.2	584	57.7	1482	55.7	
MI	Yes	947	25.8	221	21.8	726	27.3	12.96
	No	2678	72.9	780	77.0	1898	71.4	
	Missing	48	1.3	12	1.2	36	1.4	

© 2023 Mourad A et al. *JAMA Network Open.*

Variable	Level	Overall (N=3673)		Control (N=1013)		Dexamethasone (N=2660)		Std Diff Score*
Diabetes	Yes	2101	57.2	537	53.0	1564	58.8	11.67
	No	1572	42.8	476	47.0	1096	41.2	
Pre-Diabetes	Yes	39	1.1	11	1.1	28	1.1	0.32
	No	3634	98.9	1002	98.9	2632	98.9	
Obesity	Yes	1761	47.9	440	43.4	1321	49.7	12.51
	No	1912	52.1	573	56.6	1339	50.3	
Asthma	Yes	294	8.0	76	7.5	218	8.2	2.58
	No	3379	92.0	937	92.5	2442	91.8	
Autoimmune	Yes	305	8.3	105	10.4	200	7.5	9.99
	No	3368	91.7	908	89.6	2460	92.5	
COPD	Yes	1111	30.2	310	30.6	801	30.1	1.06
	No	2562	69.8	703	69.4	1859	69.9	
Sarcoidosis	Yes	3	0.1	1	0.1	2	0.1	0.80
	No	3670	99.9	1012	99.9	2658	99.9	
ILD	Yes	23	0.6	4	0.4	19	0.7	4.30
	No	3650	99.4	1009	99.6	2641	99.3	
Stroke	Yes	279	7.6	89	8.8	190	7.1	6.06
	No	3346	91.1	912	90.0	2434	91.5	
	Missing	48	1.3	12	1.2	36	1.4	
PE	Yes	195	5.3	47	4.6	148	5.6	4.27
	No	3430	93.4	954	94.2	2476	93.1	
	Missing	48	1.3	12	1.2	36	1.4	

© 2023 Mourad A et al. *JAMA Network Open.*

Variable	Level	Overall (N=3673)		Control (N=1013)		Dexamethasone (N=2660)		Std Diff Score*
HIV/AIDS	Yes	19	0.5	5	0.5	14	0.5	0.46
	No	3654	99.5	1008	99.5	2646	99.5	
Lymphoma/Leukemia	Yes	46	1.3	14	1.4	32	1.2	1.59
	No	3627	98.7	999	98.6	2628	98.8	
Solid tumors	Yes	198	5.4	56	5.5	142	5.3	0.84
	No	3475	94.6	957	94.5	2518	94.7	
CKD	Yes	2964	80.7	774	76.4	2190	82.3	14.68
	No	709	19.3	239	23.6	470	17.7	
Prior Dialysis	Yes	12	0.3	7	0.7	5	0.2	7.61
	No	3661	99.7	1006	99.3	2655	99.8	
Prior Revascularization	Yes	434	11.8	131	12.9	303	11.4	4.76
	No	3200	87.1	871	86.0	2329	87.6	
	Missing	39	1.1	11	1.1	28	1.1	
Charlson Comorbidity Index	2: 2+ comorbidities	2429	66.1	658	65.0	1771	66.6	11.15
	1: 1 comorbidity	769	20.9	199	19.6	570	21.4	
	0: 0 conditions	427	11.6	144	14.2	283	10.6	
	Missing	48	1.3	12	1.2	36	1.4	
Baseline Spo2++ /ranks	Median	3348	89.0	967	92.0	2381	88.0	53.85
	25th		80.0		86.0		79.0	
	75th		94.0		96.0		92.0	
	Mean		86.4		89.6		85.1	
	STD		9.6		8.4		9.7	
	Min		60.0		60.0		60.0	
	Max		100.0		100.0		100.0	
	Missing(%)		8.8		4.5		10.5	

© 2023 Mourad A et al. *JAMA Network Open.*

Variable	Level	Overall (N=3673)		Control (N=1013)		Dexamethasone (N=2660)		Std Diff Score*
Spo2++ /ranks	Median	3368	88.0	975	91.0	2393	87.0	62.26
	25th		80.0		87.0		79.0	
	75th		93.0		95.0		92.0	
	Mean		86.1		89.8		84.6	
	STD		9.3		7.5		9.6	
	Min		60.0		60.0		60.0	
	Max		100.0		100.0		100.0	
	Missing(%)		8.3		3.8		10.0	
FiO2++ /ranks	Median	3670	100.0	1013	80.0	2657	100.0	88.57
	25th		70.0		50.0		100.0	
	75th		100.0		100.0		100.0	
	Mean		86.5		73.2		91.5	
	STD		21.7		25.6		17.5	
	Min		21.0		21.0		28.0	
	Max		100.0		100.0		100.0	
	Missing(%)		0.1		0.0		0.1	
S/F ratio++ /ranks	Median	3365	0.9	975	1.2	2390	0.9	90.63
	25th		0.8		0.9		0.8	
	75th		1.3		1.9		1.0	
	Mean		1.1		1.4		1.0	
	STD		0.5		0.7		0.4	
	Min		0.6		0.6		0.6	
	Max		4.6		4.6		3.4	
	Missing(%)		8.4		3.8		10.2	
eGFR++ /ranks	Median	3103	64.0	843	65.6	2260	63.6	4.88
	25th		35.5		35.2		35.6	
	75th		105.6		111.2		103.5	
	Mean		67.9		69.3		67.4	

© 2023 Mourad A et al. *JAMA Network Open.*

Variable	Level	Overall (N=3673)		Control (N=1013)		Dexamethasone (N=2660)		Std Diff Score*
	STD		37.3		38.3		36.9	
	Min		3.6		4.7		3.6	
	Max		120.0		120.0		120.0	
	Missing(%)		15.5		16.8		15.0	
Time since admission++ /ranks	Median	3673	1.0	1013	1.0	2660	1.0	72.93
	25th		1.0		1.0		1.0	
	75th		1.0		3.0		1.0	
	Mean		1.5		2.3		1.2	
	STD		1.7		2.7		1.0	
	Min		1.0		1.0		1.0	
	Max		20.0		20.0		18.0	
	Missing(%)		0.0		0.0		0.0	
ICU	Yes	3436	93.5	981	96.8	2455	92.3	20.17
	No	237	6.5	32	3.2	205	7.7	
Anticoagulants (prophylactic)	Yes	1590	43.3	451	44.5	1139	42.8	3.43
	No	2083	56.7	562	55.5	1521	57.2	
Anticoagulants (treatment)	Yes	1087	29.6	338	33.4	749	28.2	11.30
	No	2586	70.4	675	66.6	1911	71.8	
Remdesivir	Yes	1032	28.1	208	20.5	824	31.0	24.06
	No	2641	71.9	805	79.5	1836	69.0	
Vasopressors	Yes	793	21.6	263	26.0	530	19.9	14.40
	No	2880	78.4	750	74.0	2130	80.1	
Tociluzimab	Yes	127	3.5	26	2.6	101	3.8	7.01
	No	3546	96.5	987	97.4	2559	96.2	

© 2023 Mourad A et al. *JAMA Network Open.*

Variable	Level	Overall (N=3673)		Control (N=1013)		Dexamethasone (N=2660)		Std Diff Score*
Baricitinib	Yes	52	1.4	21	2.1	31	1.2	7.20
	No	3621	98.6	992	97.9	2629	98.8	
Bamlanivimab/Etesevimab	No	3673	100.0	1013	100.0	2660	100.0	.
Casirivimab/Imdevimab	No	3673	100.0	1013	100.0	2660	100.0	.
Azithromycin	Yes	1681	45.8	334	33.0	1347	50.6	36.41
	No	1992	54.2	679	67.0	1313	49.4	
Antibiotics	Yes	3162	86.1	847	83.6	2315	87.0	9.67
	No	511	13.9	166	16.4	345	13.0	

* Standardized difference scores measure the effect size between Control and Dexamethasone (All Standardized difference scores have been multiplied by 100).
++ /ranks indicates standardized difference score was calculated using rank statistics for the row variable.

© 2023 Mourad A et al. *JAMA Network Open.*

eFigure 5. Unadjusted Connect-S Plot for No Supplemental Oxygen Cohort

ASMD <0.10 is very good balance and <0.20 is adequate. Subgroups with ASMD > 0.20 should be interpreted with caution.

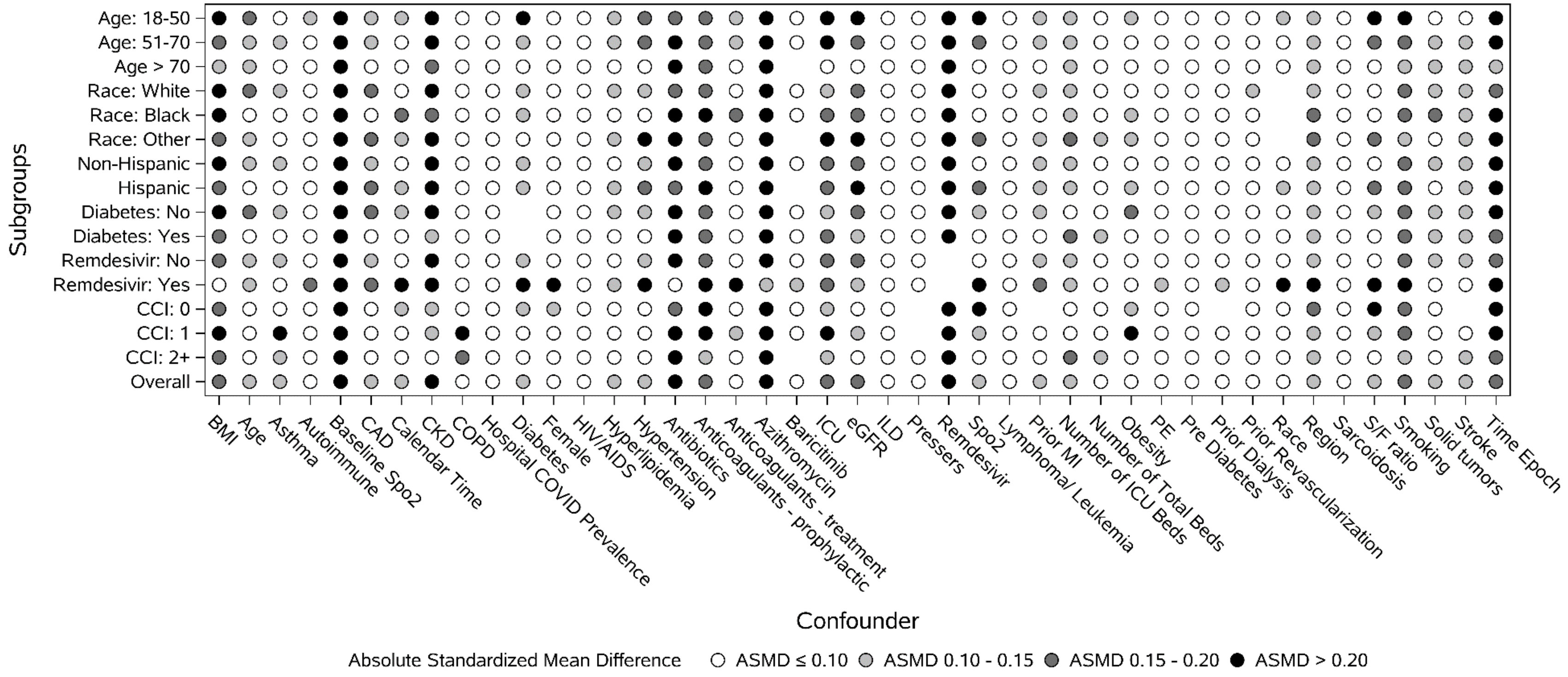

© 2023 Mourad A et al. *JAMA Network Open.*

eFigure 6. IPW-Logistic Connect-S Plot for No Supplemental Oxygen Cohort

After adjustment by inverse probability of treatment weighting with logistic regression estimation of the propensity score model (Standard method).

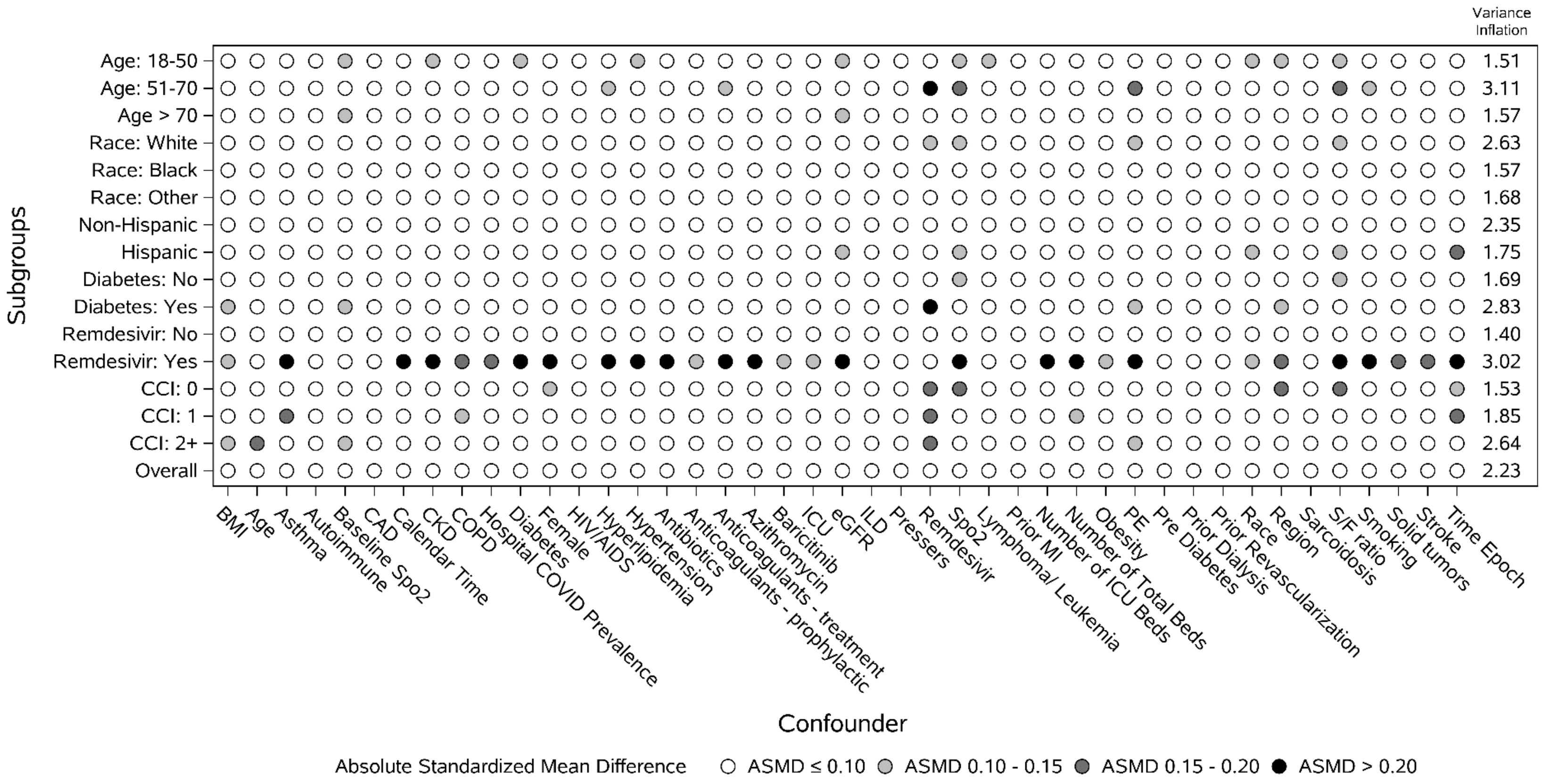

© 2023 Mourad A et al. *JAMA Network Open.*

eFigure 7. OW Adjusted Connect-S Plot for No Supplemental Oxygen Cohort

After adjustment by overlap probability of treatment weighting with LASSO estimation of the propensity score model (improved method).

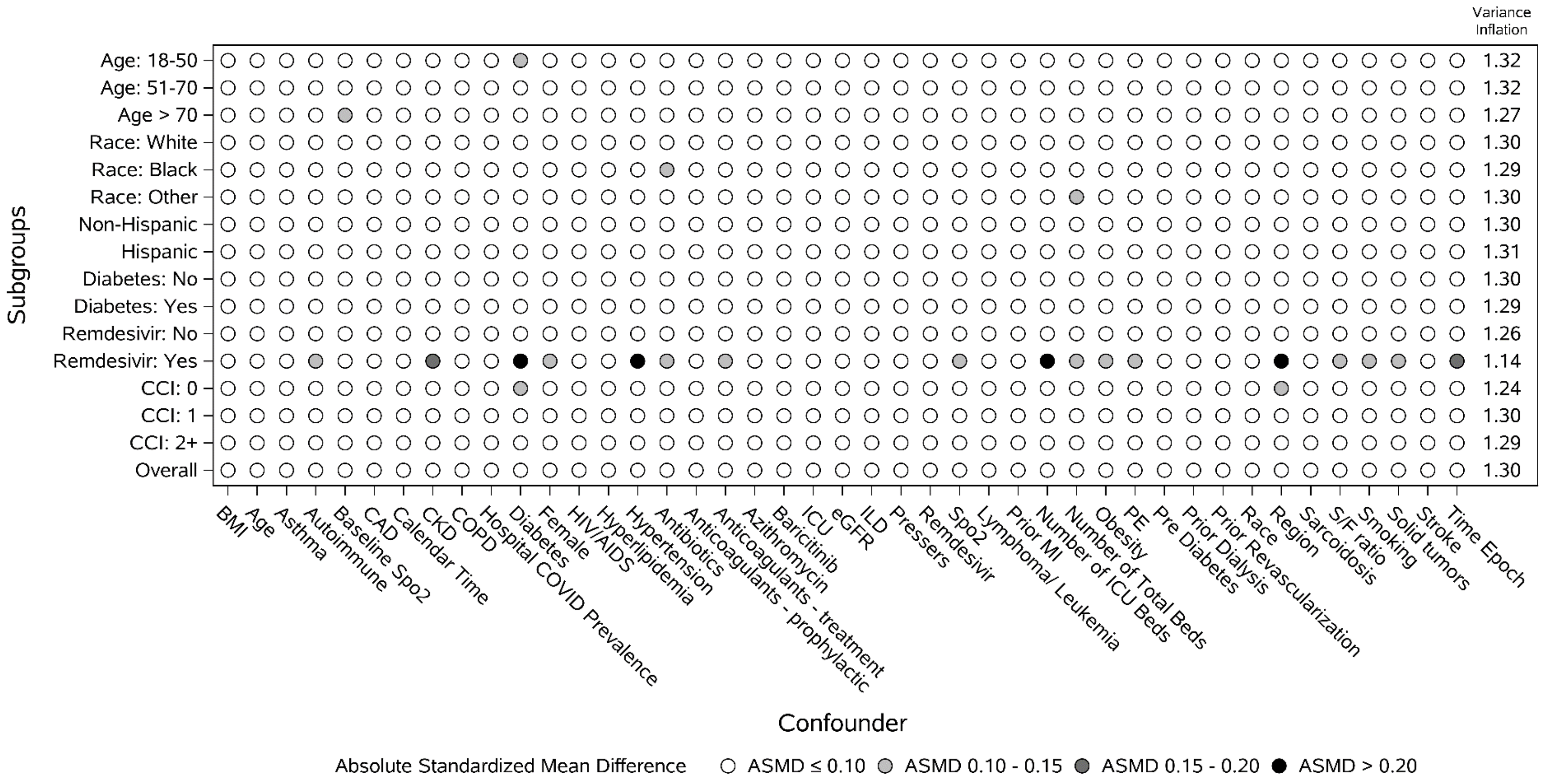

© 2023 Mourad A et al. *JAMA Network Open.*

eFigure 8. Unadjusted Connect-S Plot for Supplemental Oxygen Cohort

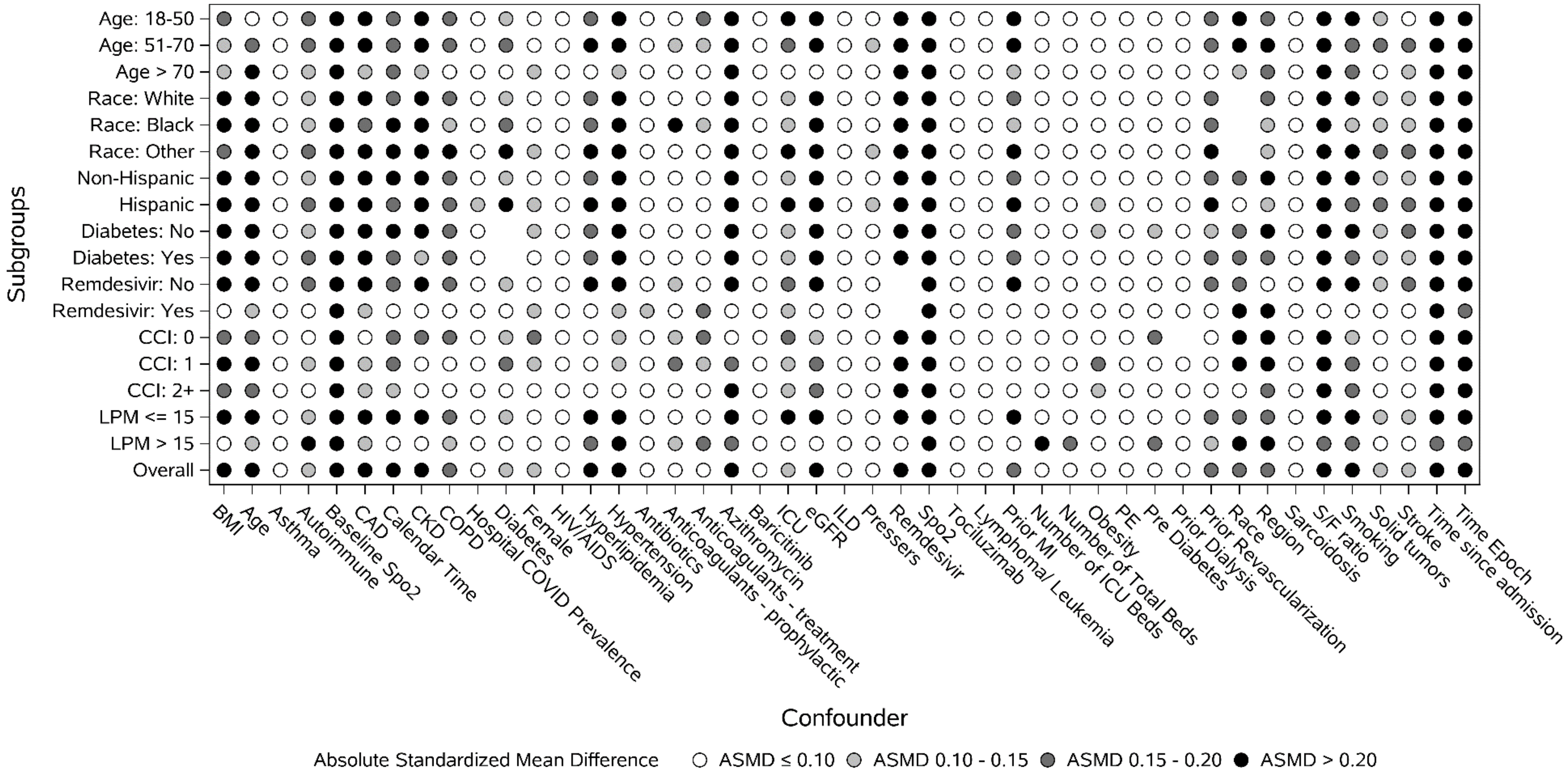

© 2023 Mourad A et al. *JAMA Network Open.*

eFigure 9. IPW-Logistic Connect-S Plot for Supplemental Oxygen Cohort

After adjustment by inverse probability of treatment weighting with logistic regression estimation of the propensity score model (Standard method).

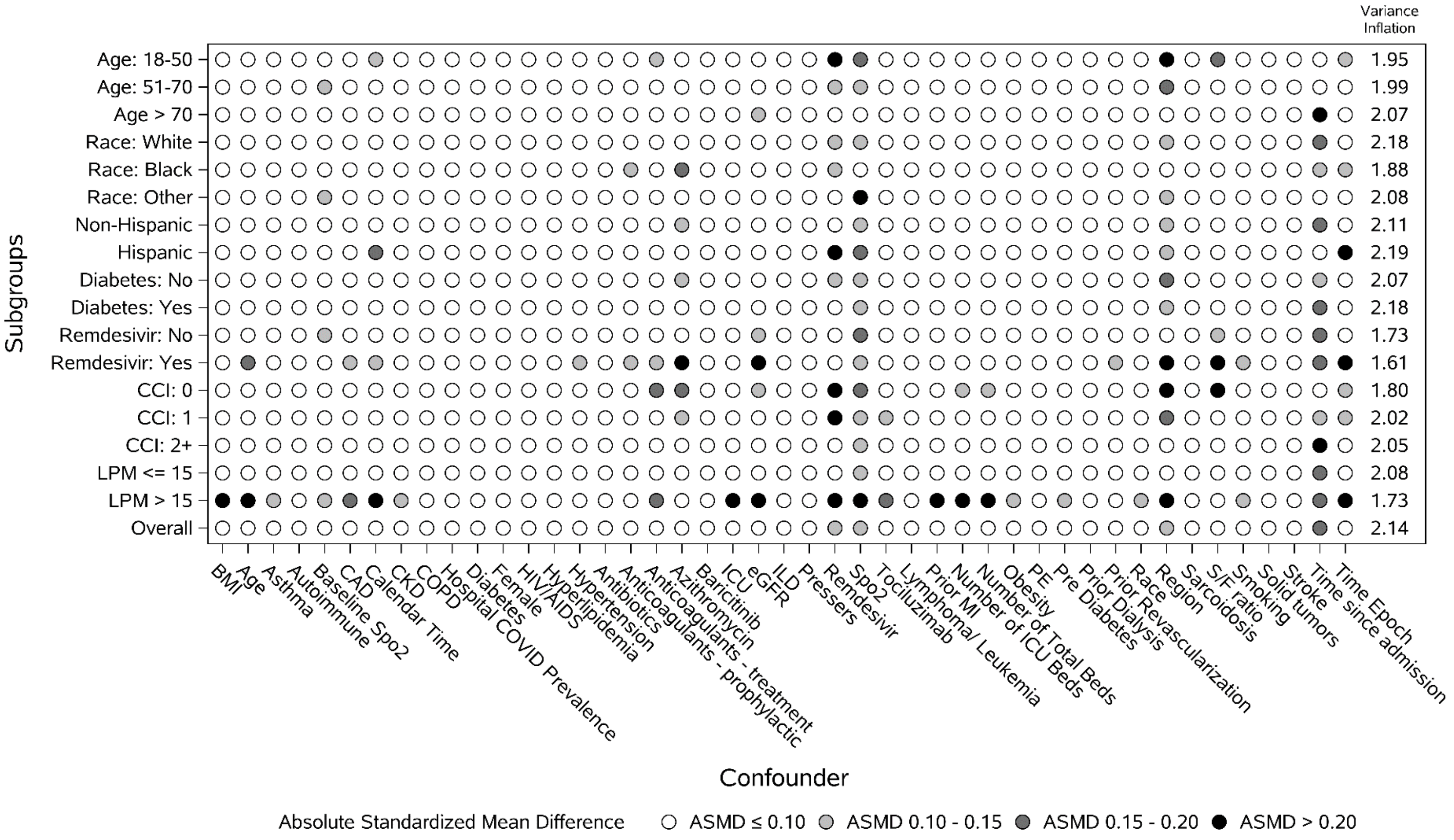

© 2023 Mourad A et al. *JAMA Network Open.*

eFigure 10. OW Adjusted Connect-S Plot for Supplemental Oxygen Cohort

After adjustment by overlap probability of treatment weighting with LASSO estimation of the propensity score model (improved method).

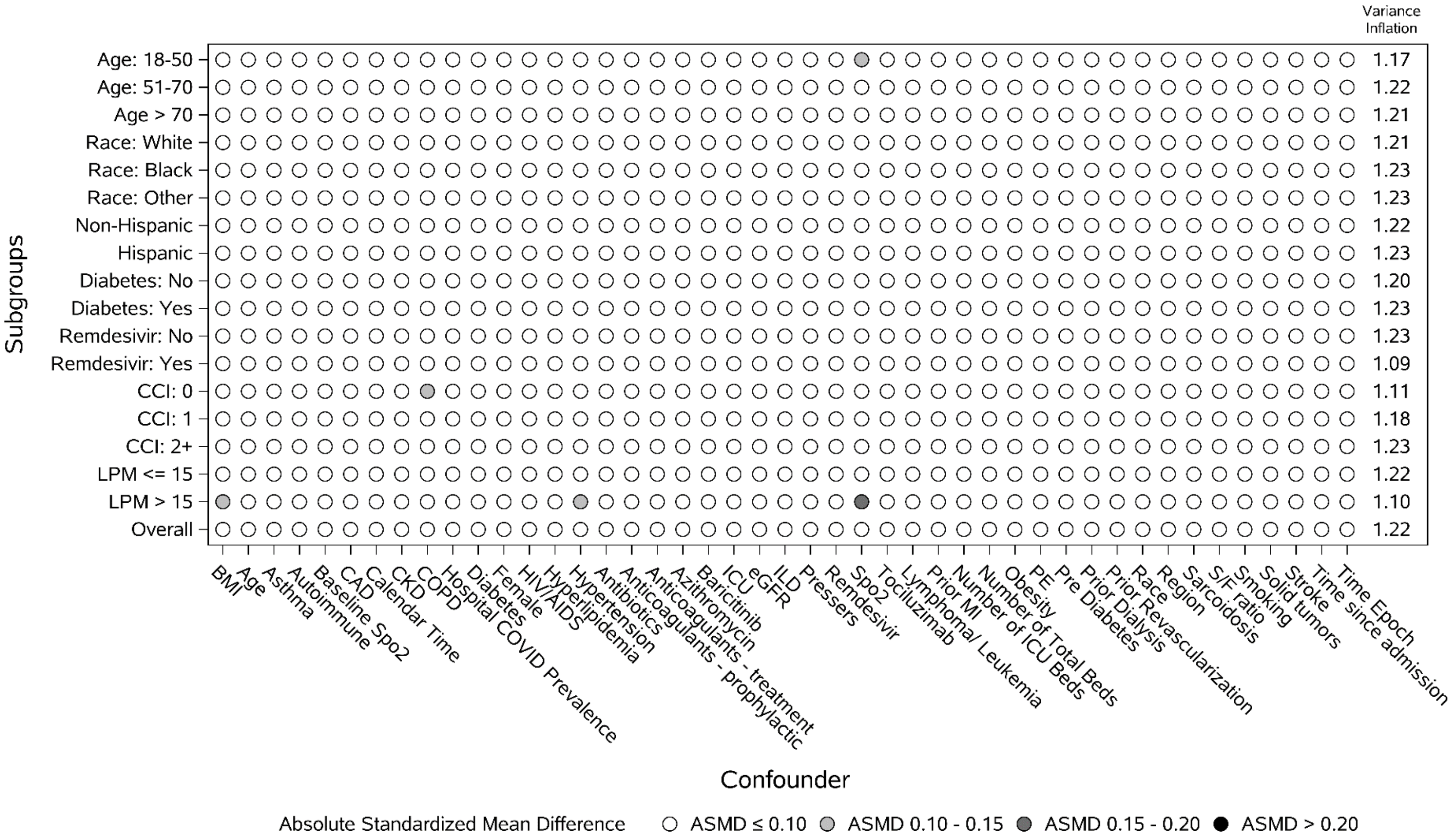

© 2023 Mourad A et al. *JAMA Network Open.*

eFigure 11. Unadjusted Connect-S Plot for NIPPV Cohort

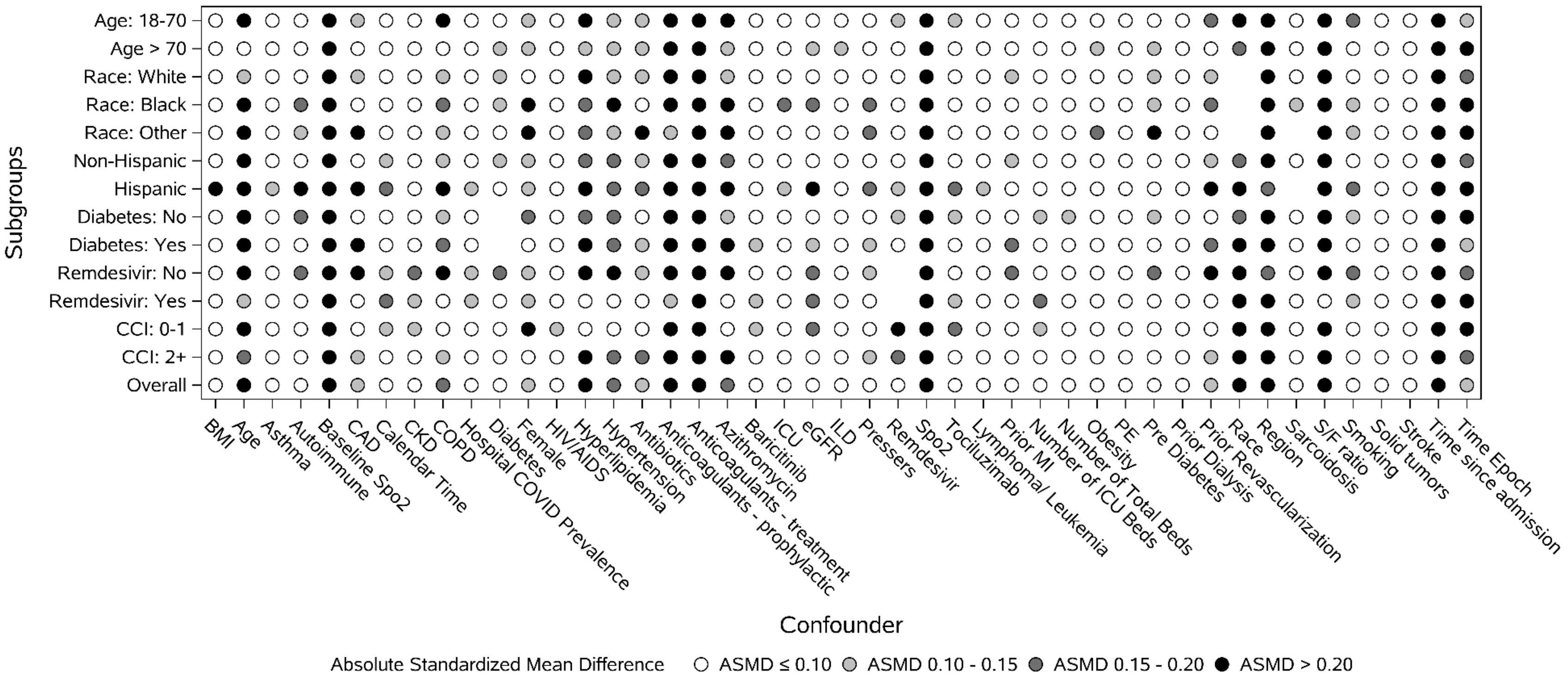

© 2023 Mourad A et al. *JAMA Network Open.*

eFigure 12. IPW-Logistic Connect-S Plot for NIPPV Cohort

After adjustment by inverse probability of treatment weighting with logistic regression estimation of the propensity score model (Standard method).

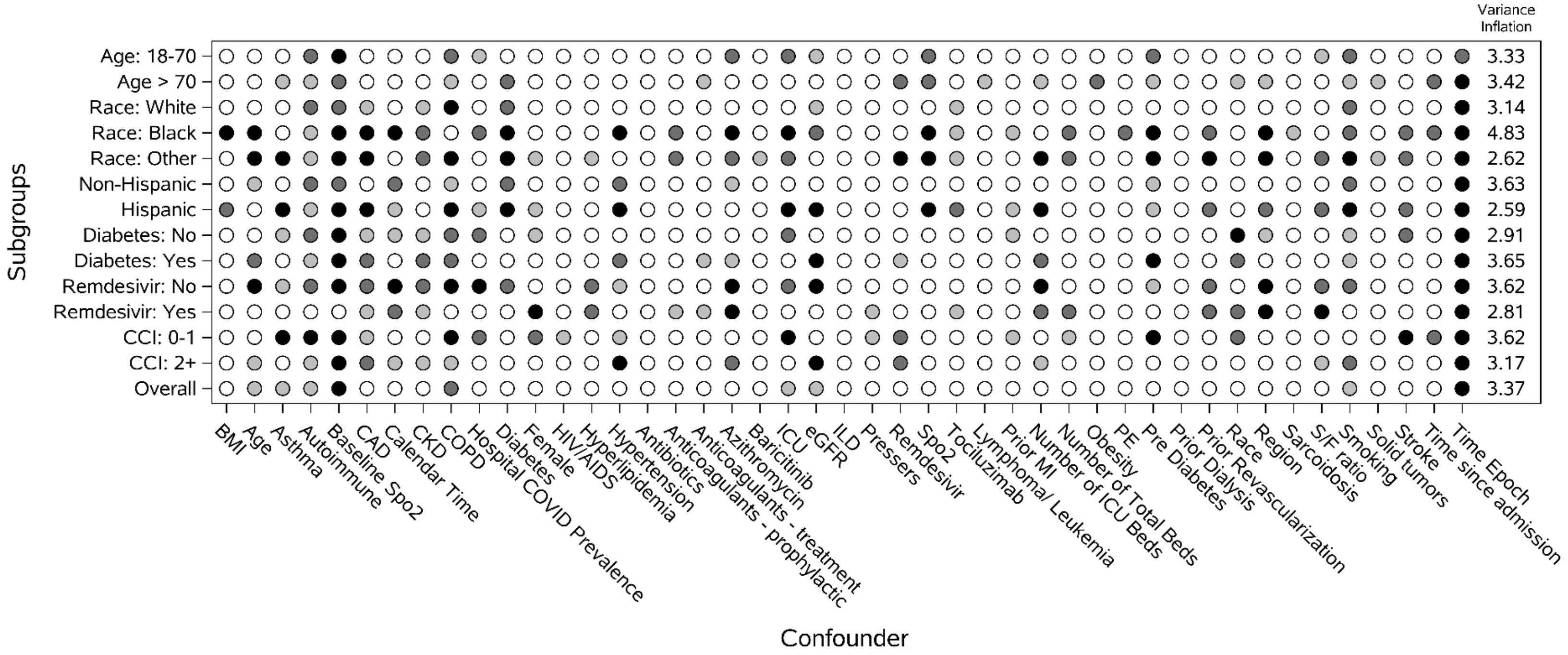

© 2023 Mourad A et al. *JAMA Network Open.*

eFigure 13. OW Adjusted Connect-S Plot for NIPPV Cohort

After adjustment by overlap probability of treatment weighting with LASSO estimation of the propensity score model (improved method).

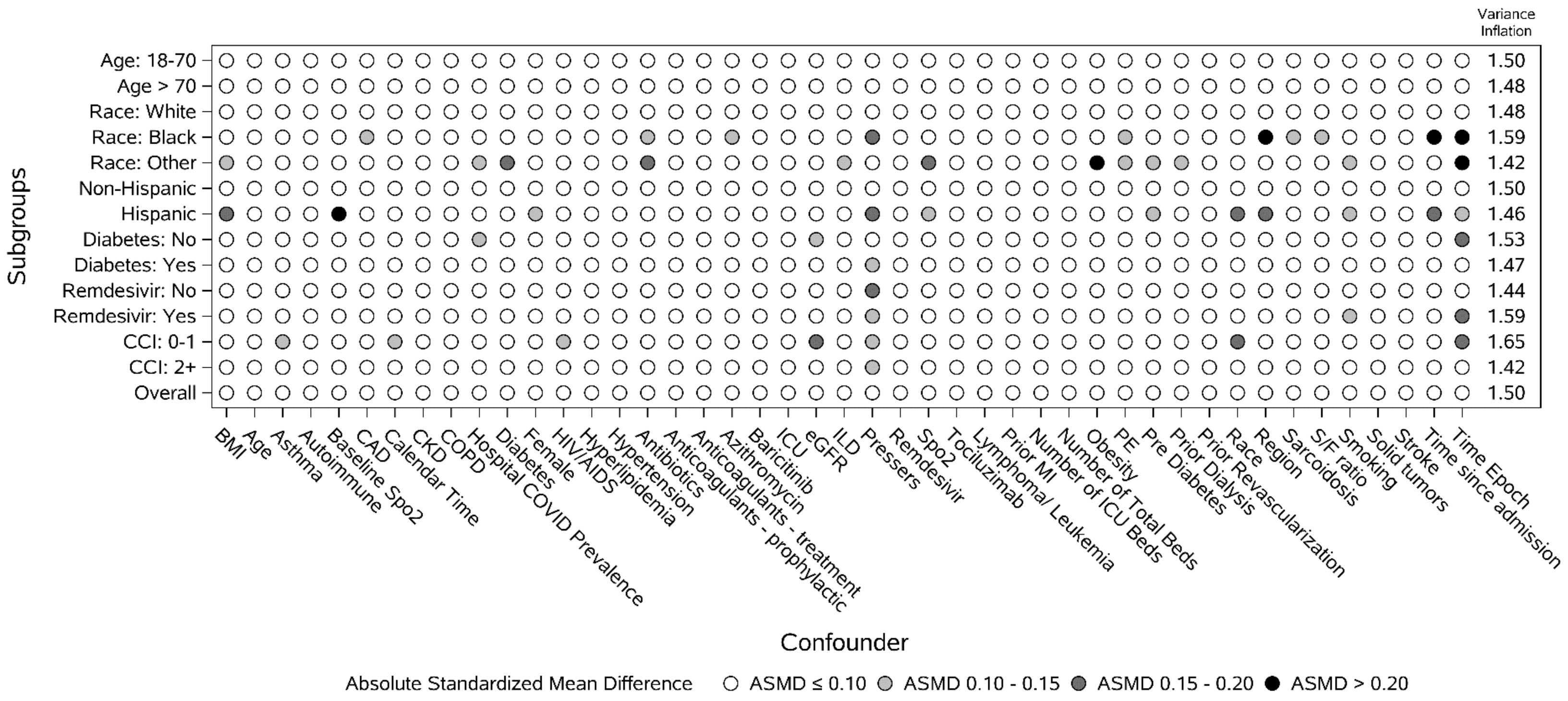

© 2023 Mourad A et al. *JAMA Network Open.*

eFigure 14. Unadjusted Connect-S Plot for MV or ECMO Cohort

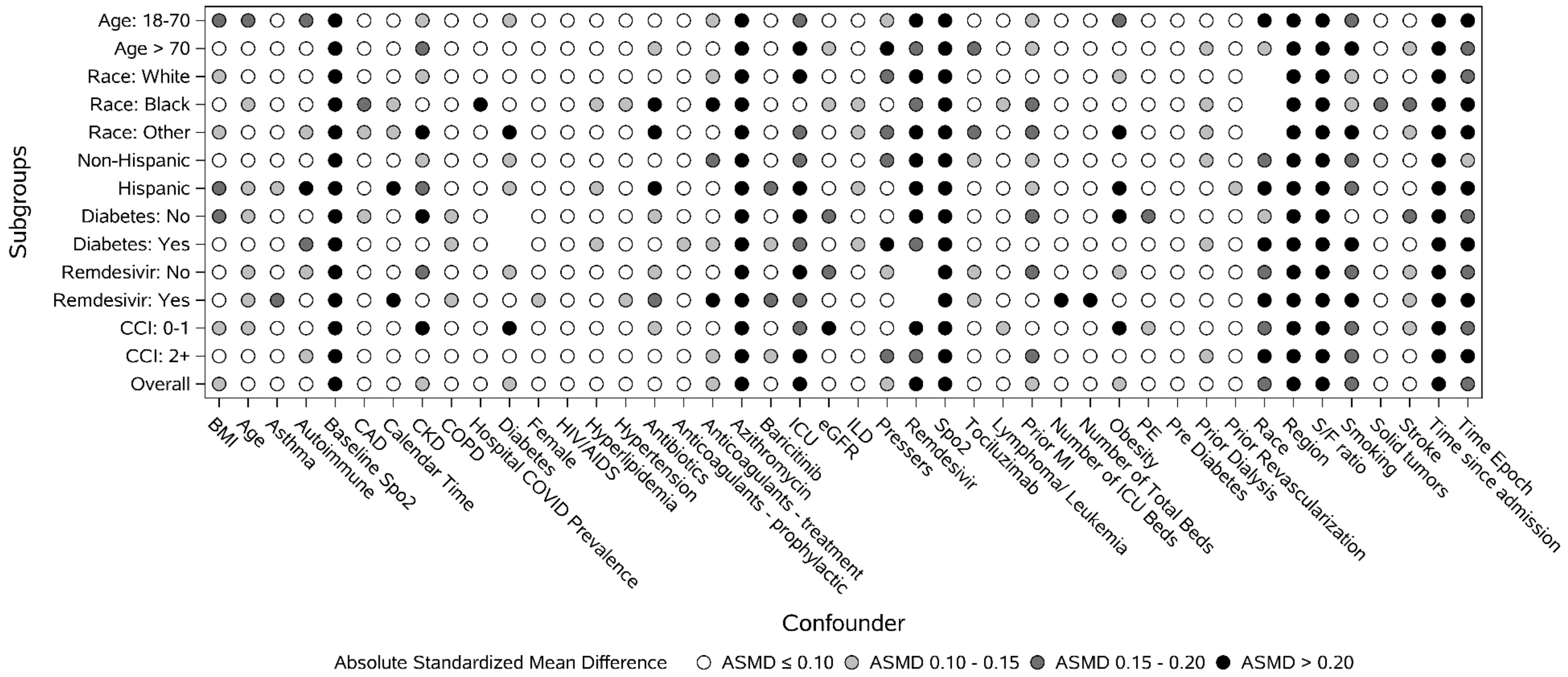

© 2023 Mourad A et al. *JAMA Network Open.*

eFigure 15. IPW-Logistic Connect-S Plot for MV or ECMO Cohort

After adjustment by inverse probability of treatment weighting with logistic regression estimation of the propensity score model (Standard method).

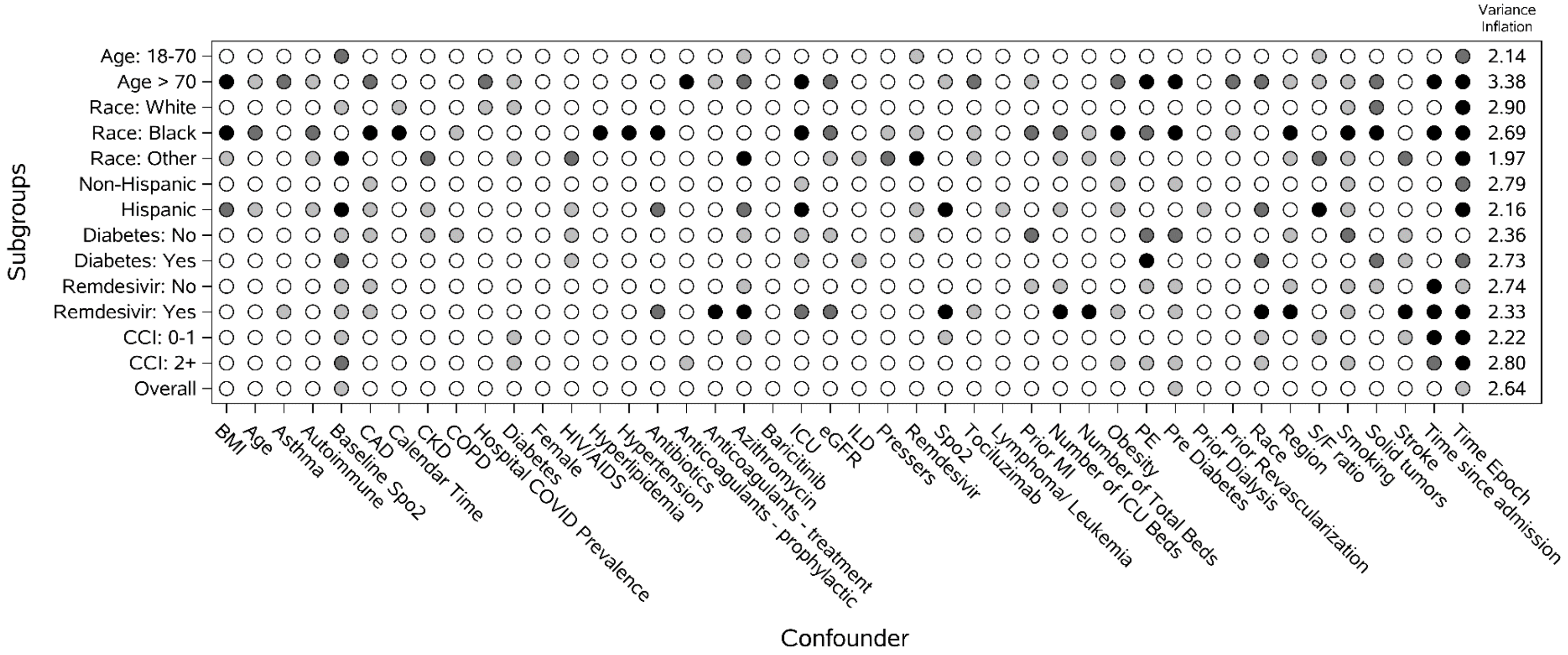

© 2023 Mourad A et al. *JAMA Network Open.*

eFigure 16. OW Adjusted Connect-S Plot for MV or ECMO Cohort

After adjustment by overlap probability of treatment weighting with LASSO estimation of the propensity score model (improved method).

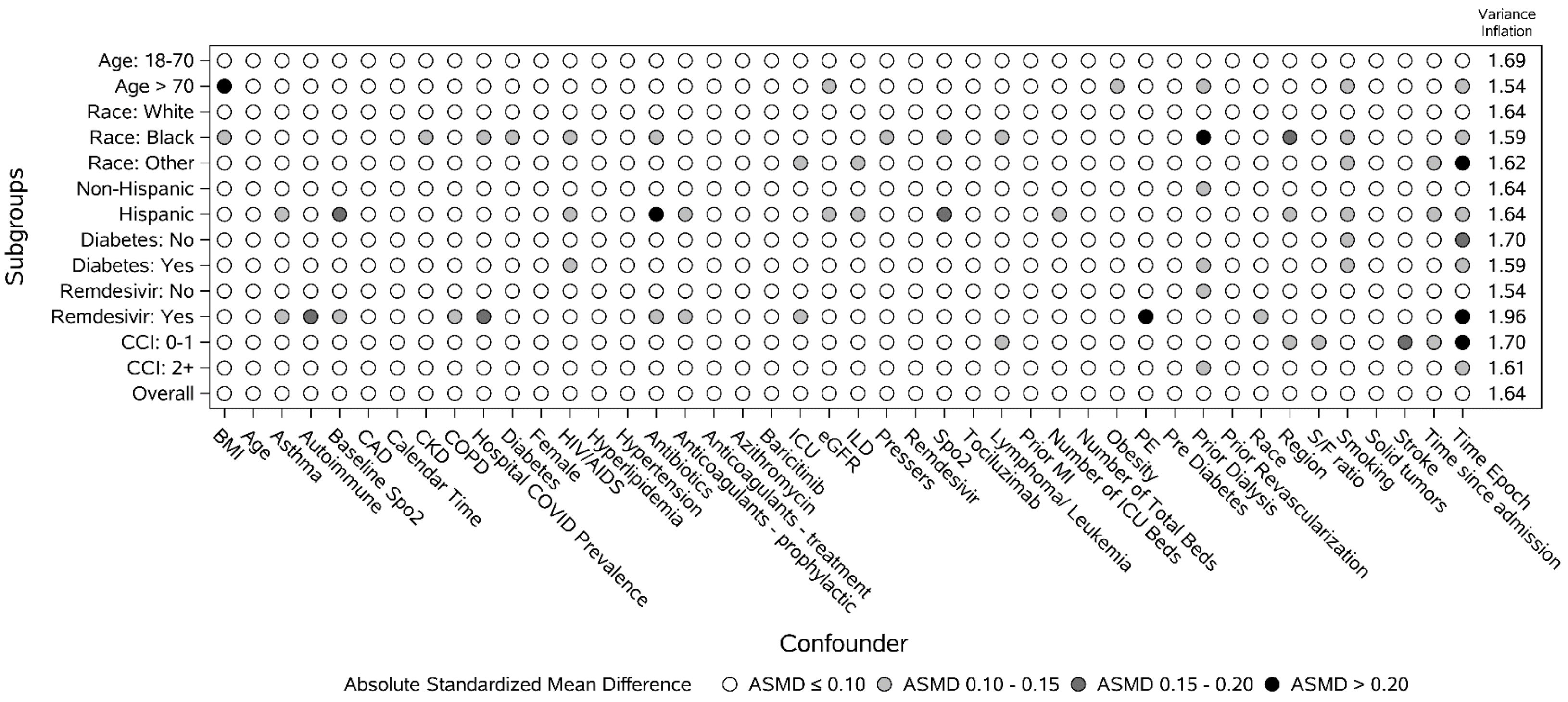

© 2023 Mourad A et al. *JAMA Network Open.*

eFigure 17. Histogram of the Propensity Score Distribution Overlaid by Treatment After Overlap Weighting for No Supplemental Oxygen Cohort

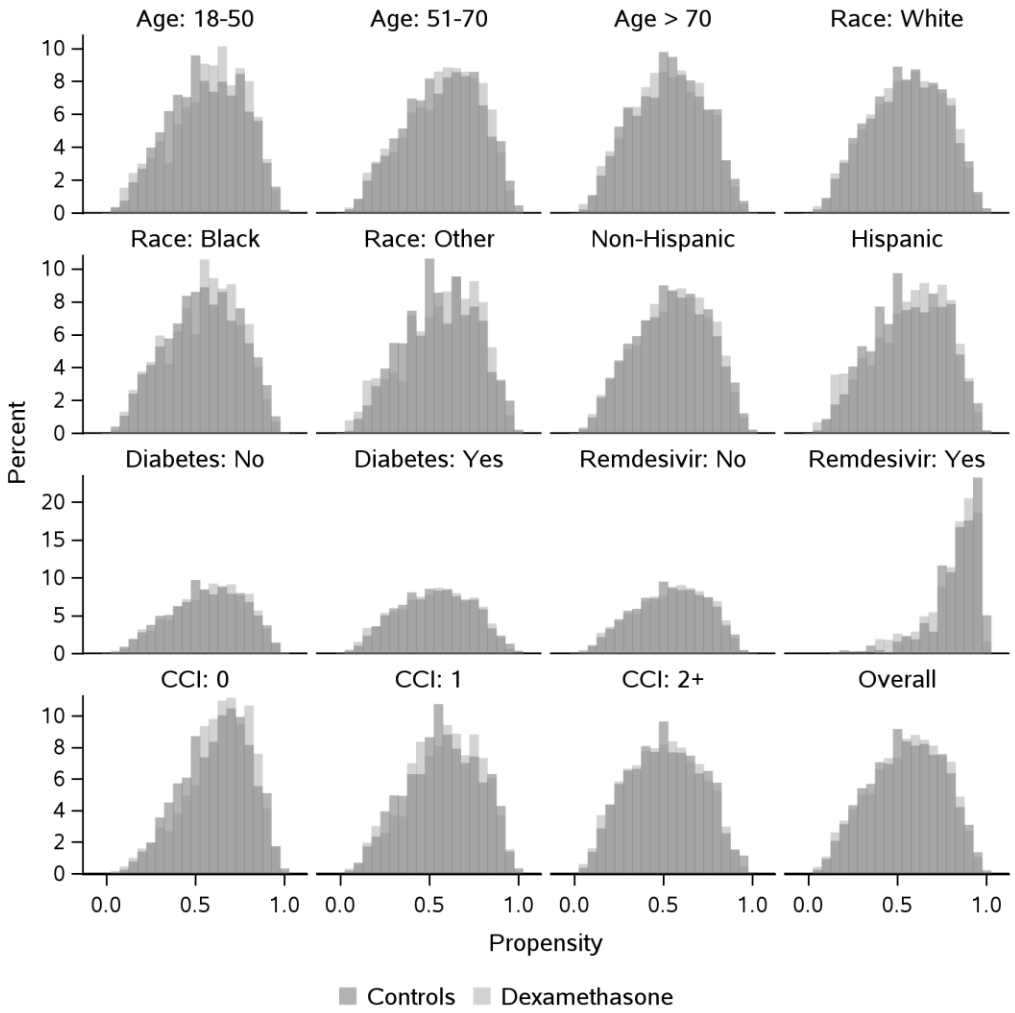

© 2023 Mourad A et al. *JAMA Network Open.*

eFigure 18. Histogram of the Propensity Score Distribution Overlaid by Treatment After Overlap Weighting for Supplemental Oxygen Cohort

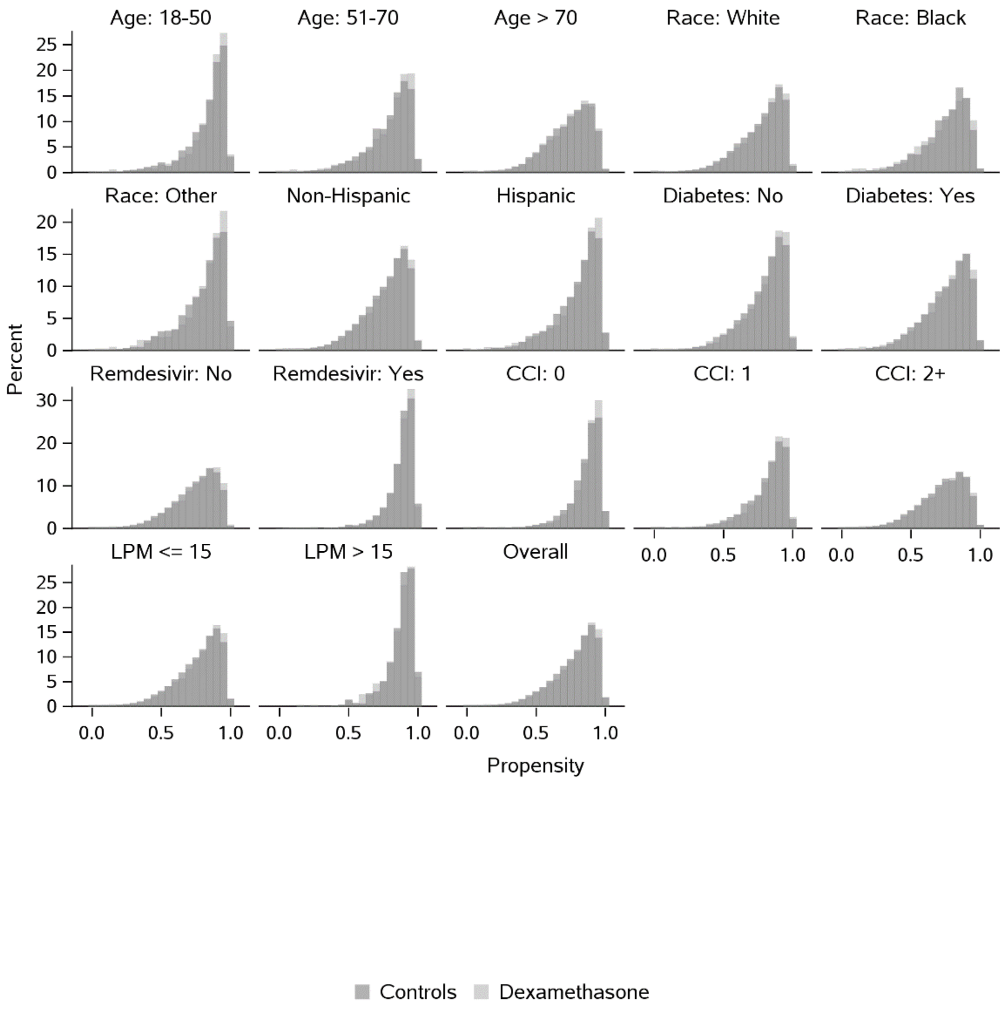

© 2023 Mourad A et al. *JAMA Network Open.*

eFigure 19. Histogram of the Propensity Score Distribution Overlaid by Treatment After Overlap Weighting for NIPPV Cohort

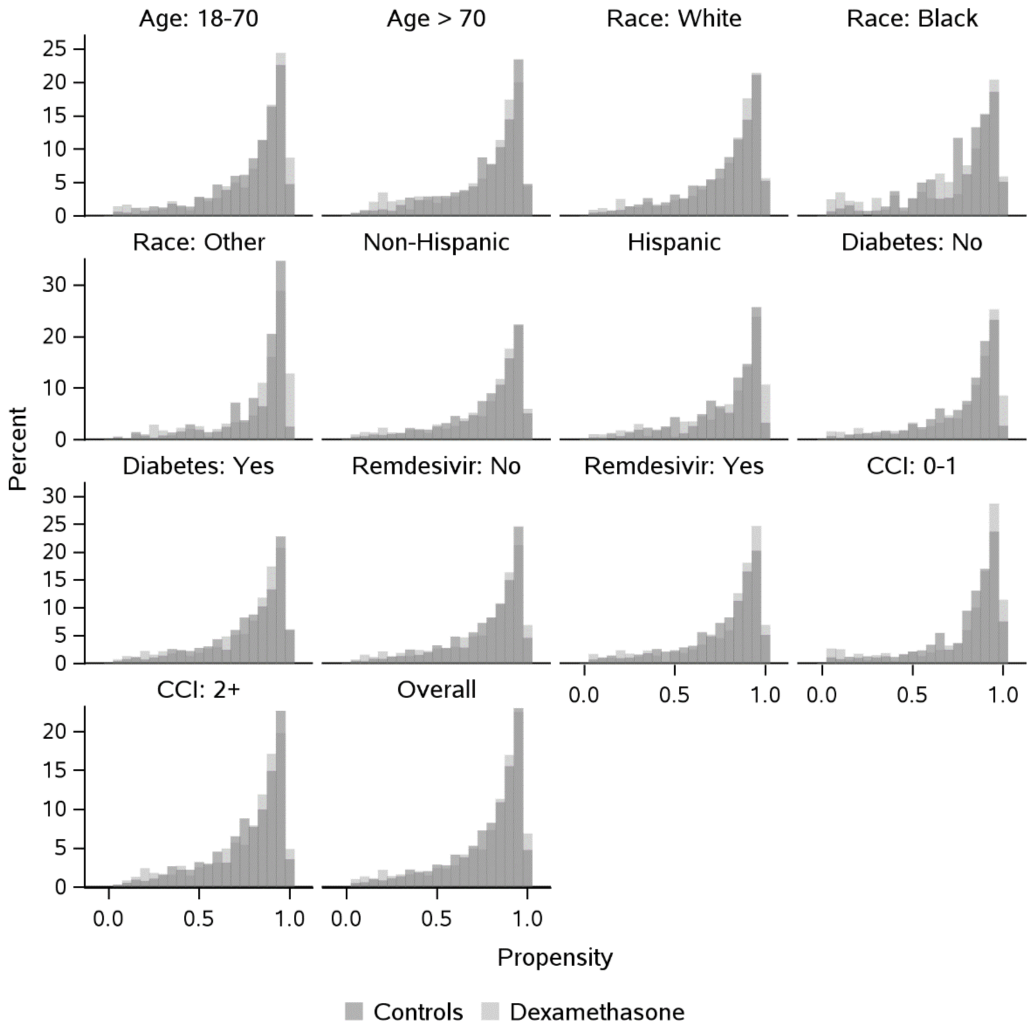

© 2023 Mourad A et al. *JAMA Network Open.*

eFigure 20. Histogram of the Propensity Score Distribution Overlaid by Treatment After Overlap Weighting for MV or ECMO Cohort

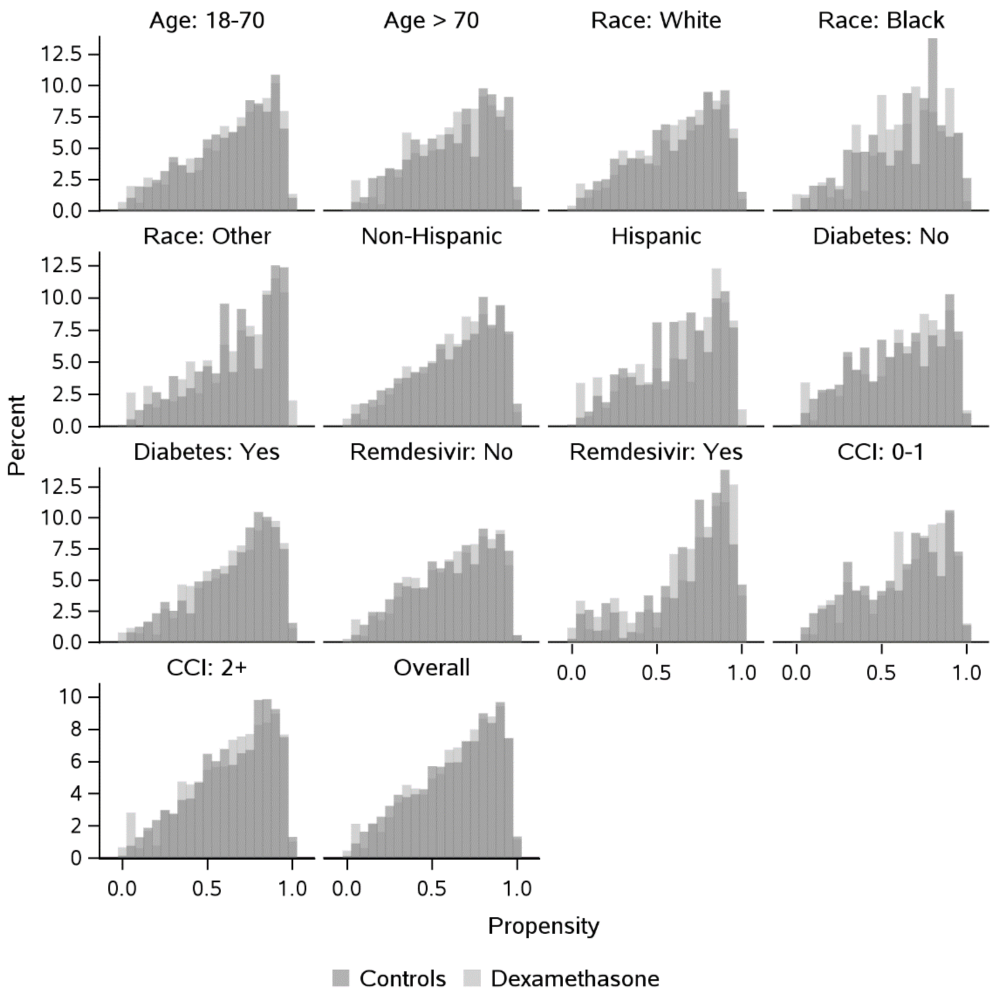

© 2023 Mourad A et al. *JAMA Network Open.*

eAppendix 3. Handling of Missing Data and Multiple Imputation

Missing data was not imputed for univariable descriptive tables and rates of missingness are reported in Supplement E. Community variables (bed size and ICU bed size) were imputed to the median value. Missing data for the covariates that vary over the course of hospitalization was imputed as follows: Missing at baseline (day 0) was imputed to the value measured at day 1 if available. This reflects the possibility that admissions late in the day may not have data added until the next day. The baseline value was not imputed with data beyond day 1. Intermittent missing data was imputed with the last value carried forward method to reflect the assumption that clinical decisions would be based on the last measured value and thus, accurately measure confounding.

Missing data for covariates was imputed as follows. Multiple imputation was done by fully conditional specification methods with 10 imputed datasets. Imputation was done using all main effects listed as well as including both in hospital mortality and mortality/hospice endpoints. The models also accounted for oxygenation level. For all missing variables we used 30 burn-in iterations. Missing binary variables were imputed using the logistic method with a logit link. Whereas categorical variables were imputed using the generalized logit function. Lastly, continuous variables were imputed using the predictive mean matching method. The analysis of outcomes after multiple imputation was conducted in SAS PROC MI and accounted for variability introduced by imputation.

The results based on a single imputed data set and multiply imputed data set were essentially identical. We, therefore, moved forward with the single imputed data set for simplicity. For example, here is a comparison of overall results with differences in bold:

eTable 5. Adjusted Odds Ratios of Single Imputed Data Set and Multiply Imputed Data Set

	**Single imputed data set**	**Multiply imputation analysis.**
**No oxygen**	aOR 0.90, 95% CI 0.78-1.03	aOR 0.90, 95% CI 0.78-1.03
**Supplemental oxygen**	aOR **0.92**, 95% CI 0.86-0.98	aOR **0.91**, 95% CI 0.86-0.98
**NIPPV**	aOR 0.87, 95% CI **0.73**-1.04	aOR 0.87, 95% CI **0.72**-1.04
**MV/ECMO**	aOR 0.82, 95% CI 0.68-0.99	aOR 0.82, 95% CI 0.68-0.99

© 2023 Mourad A et al. *JAMA Network Open.*

eTable 6. Frequency of End Points per Cohort

**No supplemental oxygen**								
**End Point**	**Level**	**Overall (N=13040)**		**Control (N=5503)**		**Dexamethasone (N=7537)**		**P-value**
Mortality	Yes	852	6.53	438	7.96	414	5.49	<.0001
	No	12188	93.47	5065	92.04	7123	94.51	
Mortality/Hospice	Yes	1204	9.23	661	12.01	543	7.20	<.0001
	No	11836	90.77	4842	87.99	6994	92.80	

**Supplemental oxygen**								
**End Point**	**Level**	**Overall (N=56368)**		**Control (N=7789)**		**Dexamethasone (N=48579)**		**P-value**
Mortality	Yes	8335	14.79	1357	17.42	6978	14.36	<.0001
	No	48033	85.21	6432	82.58	41601	85.64	
Mortality/Hospice	Yes	9840	17.46	1748	22.44	8092	16.66	<.0001
	No	46528	82.54	6041	77.56	40487	83.34	

**Non-invasive positive pressure ventilation**								
**End Point**	**Level**	**Overall (N=7618)**		**Control (N=792)**		**Dexamethasone (N=6826)**		**P-value**
Mortality	Yes	3574	46.92	384	48.48	3190	46.73	0.3498
	No	4044	53.08	408	51.52	3636	53.27	
Mortality/Hospice	Yes	3841	50.42	421	53.16	3420	50.10	0.1037
	No	3777	49.58	371	46.84	3406	49.90	

**Mechanical ventilation or ECMO**								
**End Point**	**Level**	**Overall (N=3673)**		**Control (N=1013)**		**Dexamethasone (N=2660)**		**P-value**
Mortality	Yes	2068	56.30	546	53.90	1522	57.22	0.0700
	No	1605	43.70	467	46.10	1138	42.78	
Mortality/Hospice	Yes	2195	59.76	586	57.85	1609	60.49	0.1447
	No	1478	40.24	427	42.15	1051	39.51	

© 2023 Mourad A et al. *JAMA Network Open.*

eTable 7. Unadjusted Analysis of In-Hospital Mortality or Discharge to Hospice

<table>
<tr><th></th><th>No supplemental oxygen</th><th>Supplemental oxygen</th><th>NIPPV</th><th>MV/ECMO</th></tr>
<tr><th>Variable</th><th>OR (95% CI)</th><th>OR (95% CI)</th><th>OR (95% CI)</th><th>OR (95% CI)</th></tr>
<tr><td>Age</td><td></td><td></td><td></td><td></td></tr>
<tr><td>18-50</td><td>0.49 (0.30, 0.78)</td><td>0.81 (0.64, 1.02)</td><td rowspan="2">0.89 (0.73, 1.08)</td><td rowspan="2">1.11 (0.93, 1.32)</td></tr>
<tr><td>51-70</td><td>0.55 (0.44, 0.70)</td><td>0.81 (0.72, 0.90)</td></tr>
<tr><td>> 70</td><td>0.65 (0.56, 0.76)</td><td>0.84 (0.77, 0.90)</td><td>1.03 (0.82, 1.31)</td><td>1.20 (0.91, 1.58)</td></tr>
<tr><td>Race</td><td></td><td></td><td></td><td></td></tr>
<tr><td>Black</td><td>0.67 (0.51, 0.88)</td><td>0.69 (0.60, 0.80)</td><td>0.99 (0.69, 1.43)</td><td>1.04 (0.72, 1.48)</td></tr>
<tr><td>White</td><td>0.55 (0.47, 0.64)</td><td>0.65 (0.61, 0.70)</td><td>0.89 (0.74, 1.06)</td><td>1.16 (0.96, 1.41)</td></tr>
<tr><td>Other</td><td>0.53 (0.39, 0.71)</td><td>0.83 (0.71, 0.96)</td><td>0.78 (0.54, 1.13)</td><td>1.12 (0.83, 1.52)</td></tr>
<tr><td>Ethnicity</td><td></td><td></td><td></td><td></td></tr>
<tr><td>Non-Hispanic</td><td>0.58 (0.50, 0.66)</td><td>0.67 (0.62, 0.71)</td><td>0.90 (0.76, 1.06)</td><td>1.04 (0.88, 1.23)</td></tr>
<tr><td>Hispanic</td><td>0.56 (0.43, 0.74)</td><td>0.80 (0.70, 0.91)</td><td>0.80 (0.57, 1.12)</td><td>1.38 (1.03, 1.85)</td></tr>
<tr><td>Diabetes</td><td></td><td></td><td></td><td></td></tr>
<tr><td>No</td><td>0.46 (0.39, 0.55)</td><td>0.64 (0.59, 0.70)</td><td>0.84 (0.66, 1.05)</td><td>1.23 (0.99, 1.52)</td></tr>
<tr><td>Yes</td><td>0.72 (0.61, 0.85)</td><td>0.77 (0.71, 0.84)</td><td>0.93 (0.77, 1.12)</td><td>1.01 (0.82, 1.23)</td></tr>
<tr><td>Remdesivir</td><td></td><td></td><td></td><td></td></tr>
<tr><td>No</td><td>0.60 (0.53, 0.68)</td><td>0.70 (0.65, 0.75)</td><td>0.96 (0.79, 1.16)</td><td>1.37 (1.16, 1.62)</td></tr>
<tr><td>Yes</td><td>0.91 (0.35, 2.37)</td><td>0.91 (0.78, 1.05)</td><td>0.78 (0.62, 0.99)</td><td>0.55 (0.40, 0.76)</td></tr>
<tr><td>CCI</td><td></td><td></td><td></td><td></td></tr>
<tr><td>0</td><td>0.74 (0.45, 1.20)</td><td>0.87 (0.70, 1.07)</td><td rowspan="2">0.88 (0.68, 1.13)</td><td rowspan="2">1.14 (0.89, 1.46)</td></tr>
<tr><td>1</td><td>0.53 (0.40, 0.71)</td><td>0.82 (0.71, 0.95)</td></tr>
<tr><td>2+</td><td>0.71 (0.61, 0.81)</td><td>0.89 (0.83, 0.95)</td><td>0.95 (0.79, 1.14)</td><td>1.09 (0.91, 1.31)</td></tr>
<tr><td>O2 Flow</td><td></td><td></td><td></td><td></td></tr>
<tr><td>Low flow</td><td></td><td>0.60 (0.57, 0.64)</td><td></td><td></td></tr>
<tr><td>High flow</td><td></td><td>0.88 (0.71, 1.08)</td><td></td><td></td></tr>
<tr><td>Overall</td><td>0.57 (0.50, 0.64)</td><td>0.69 (0.65, 0.73)</td><td>0.88 (0.76, 1.03)</td><td>1.12 (0.96, 1.29)</td></tr>
</table>

© 2023 Mourad A et al. *JAMA Network Open.*

eFigure 21. Time Epoch Sensitivity Analysis for Oatients on No Supplemental Oxygen

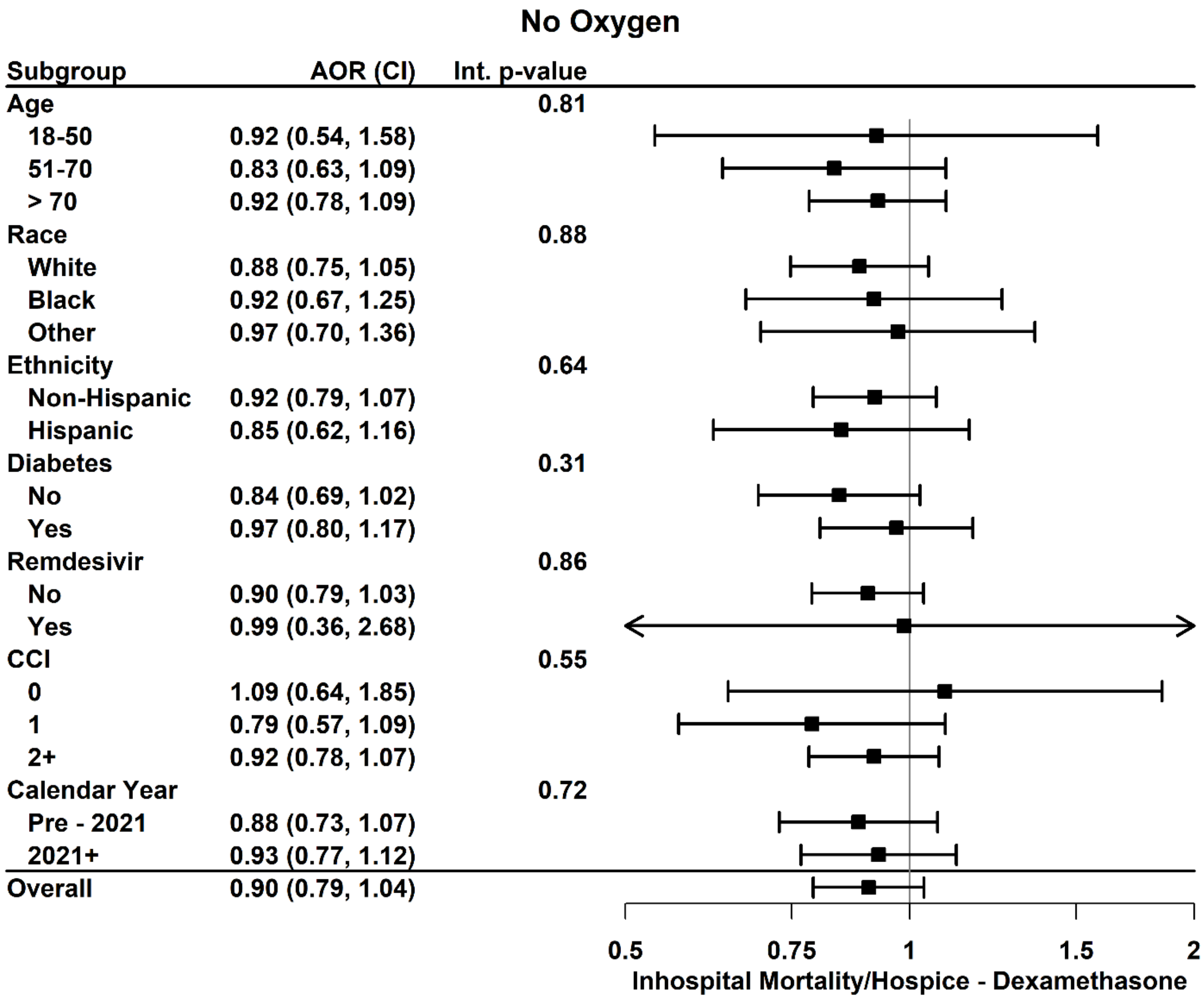

© 2023 Mourad A et al. *JAMA Network Open.*

eFigure 22. Time Epoch Sensitivity Analysis for Patients on Supplemental Oxygen

Supplemental Oxygen

Subgroup	AOR (CI)	Int. p-value
Age		0.71
18-50	0.88 (0.68, 1.14)	
51-70	0.95 (0.84, 1.08)	
> 70	0.90 (0.83, 0.98)	
Race		0.03
White	0.87 (0.80, 0.94)	
Black	0.95 (0.80, 1.12)	
Other	1.11 (0.94, 1.31)	
Ethnicity		0.11
Non-Hispanic	0.89 (0.83, 0.96)	
Hispanic	1.02 (0.88, 1.18)	
Diabetes		0.82
No	0.93 (0.84, 1.02)	
Yes	0.91 (0.83, 1.00)	
Remdesivir		0.06
No	0.89 (0.83, 0.96)	
Yes	1.04 (0.90, 1.22)	
CCI		0.87
0	0.96 (0.76, 1.20)	
1	0.93 (0.79, 1.09)	
2+	0.91 (0.84, 0.98)	
Calendar Year		0.42
Pre - 2021	0.95 (0.86, 1.04)	
2021+	0.89 (0.82, 0.98)	
Supp. O2 Flow		0.39
Low flow	0.91 (0.85, 0.97)	
High flow	1.01 (0.80, 1.26)	
Overall	0.92 (0.86, 0.98)	

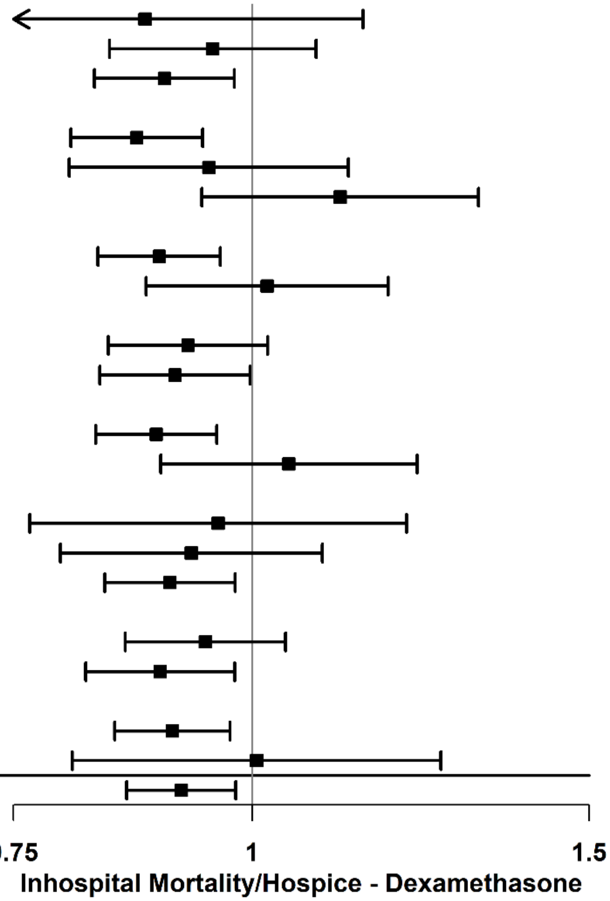

© 2023 Mourad A et al. *JAMA Network Open.*

eFigure 23. Time Epoch Sensitivity Analysis for Patients on NIPPV

NIPPV

Subgroup	AOR (CI)	Int. p-value
**Age**		0.68
18-70	0.89 (0.70, 1.14)	
> 70	0.83 (0.62, 1.10)	
**Race**		0.17
White	0.78 (0.62, 0.97)	
Black	1.25 (0.78, 1.99)	
Other	0.96 (0.62, 1.50)	
**Ethnicity**		0.52
Non-Hispanic	0.84 (0.69, 1.03)	
Hispanic	0.98 (0.65, 1.47)	
**Diabetes**		0.42
No	0.79 (0.59, 1.06)	
Yes	0.92 (0.73, 1.16)	
**Remdesivir**		0.70
No	0.85 (0.67, 1.06)	
Yes	0.91 (0.67, 1.22)	
**CCI**		0.16
0-1	1.06 (0.76, 1.47)	
2+	0.80 (0.64, 0.99)	
**Calendar Year**		0.59
Pre - 2021	0.92 (0.70, 1.21)	
2021+	0.83 (0.65, 1.06)	
**Overall**	0.87 (0.72, 1.04)	

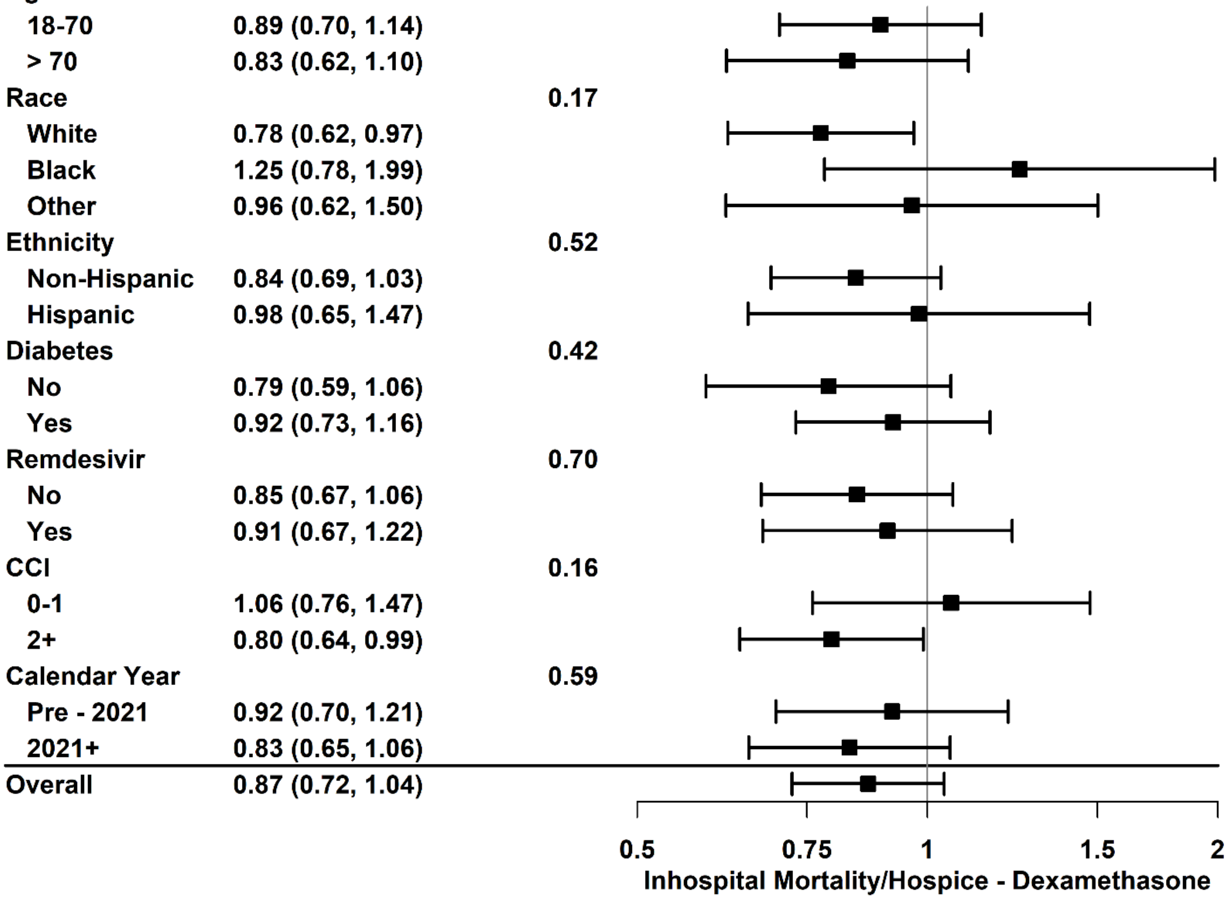

© 2023 Mourad A et al. *JAMA Network Open.*

eFigure 24. Time Epoch Sensitivity Analysis for Patients on MV/ECMO

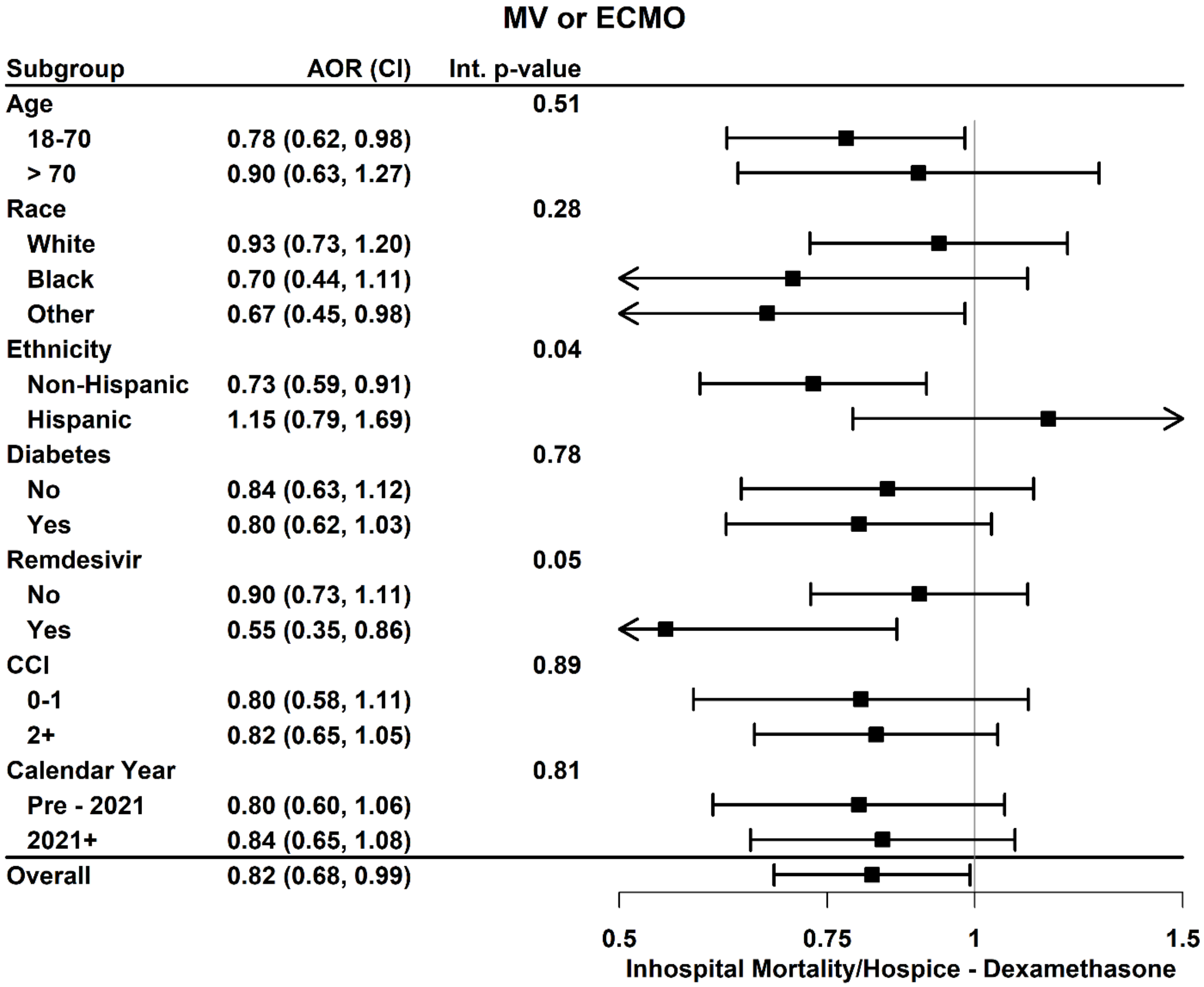

© 2023 Mourad A et al. *JAMA Network Open.*
