# Supplementary material for: Dexamethasone for Inpatients With COVID-19 in a National Cohort
Source: JAMA Netw Open. 2023 Apr 17;6(4):e238516. doi: 10.1001/jamanetworkopen.2023.8516 (PMC10111178; doi:10.1001/jamanetworkopen.2023.8516)
Supplement: Supplement 2. — Data Sharing Statement [file jamanetwopen-e238516-s002.pdf]

Data Sharing Statement

Mourad. Dexamethasone for Inpatients With COVID-19 in a National Cohort. *JAMA Netw Open*. Published April 17, 2023. doi:10.1001/jamanetworkopen.2023.8516

Data
Data available: No
